# Lignans: current trends in production through plant tissue culture, biological activities, and formulation methods

**DOI:** 10.3389/fpls.2026.1869172

**Published:** 2026-10-19

**Authors:** Miriam Ramírez-Carrillo, Francisco Cruz-Sosa, Alberto Marbán-González, Antonio Bernabé-Antonio, Mayra Antunez-Mojica, Mariana Sánchez-Ramos

**Affiliations:** 1 Facultad de Farmacia, Universidad Autónoma del Estado de Morelos, Cuernavaca, Mexico; 2 Departamento de Biotecnología, Universidad Autónoma Metropolitana unidad Iztapalapa, Ciudad de México, Mexico; 3 Grupo de Investigación DIFACQUIM, Departamento de Farmacia, Facultad de Química, Universidad Nacional Autónoma de México, Ciudad de México, Mexico; 4 Centro Universitario de Ciencias Exactas e Ingenierías, Departamento de Madera, Celulosa y Papel, Universidad de Guadalajara, Zapopan, Mexico; 5 SECIHTI-Centro de Investigaciones Químicas-IICBA, Universidad Autónoma del Estado de Morelos, Cuernavaca, Mexico; 6 Instituto de Ciencias Agropecuarias, Área Académica de Ciencias Agrícolas y Forestales, Universidad Autónoma del Estado de Hidalgo, Santiago Tulantepec, Mexico

**Keywords:** biological activities, biosynthesis, biotechnology, lignans, molecular delivery, plant tissue culture

## Abstract

Lignans constitute an important class of plant secondary metabolites, widely distributed throughout the plant kingdom and biosynthesized via the oxidative coupling of phenylpropane units. These phenolic compounds have become key components of translational medicine and the nutraceutical industry, thanks to their wide range of biological activities. These activities include antitumor, antioxidant, anti-inflammatory, antiviral, neuroprotective, cardioprotective, and hormone-modulating effects, making them strategic candidates for treating chronic degenerative diseases with high global prevalence. However, harnessing their therapeutic potential presents significant challenges, as their taxonomic distribution is limited and their concentrations are concentrated in a few plant families, thereby hindering their commercial availability. Furthermore, the stereochemical complexity of their structures, characterized by multiple chiral centers, imposes significant barriers to total chemical synthesis and reduces the economic viability of synthetic routes. This gap between growing demand and limited natural supply underscores the urgent need to explore new plant species that produce lignans. In this context, this review critically examines the main sources of lignans, including direct extraction from plant biomass, chemical synthesis, and biotechnological production through callus cultures, cell suspensions, and hairy root cultures. The bioactivities and molecular mechanisms of lignans and neolignans obtained by biotechnological methods are further explored, including analyses of species distribution and molecular targets. Finally, the review discusses the advantages and limitations of different production platforms, challenges associated with industrial-scale production, and future perspectives for the sustainable and scalable development of lignan-based products.

## 1 Introduction

Lignans constitute a broad and chemodiverse class of phenylpropanoid secondary metabolites, distributed with remarkable abundance in woody and herbaceous taxa (Cui et al., 2020; Ražná et al., 2021). Biomedical and pharmaceutical interest in lignans is largely driven by their antioxidant, antitumor, antiviral, and anti-inflammatory activities (Karimi and Rashidinejad, 2022; Plaha et al., 2022). A critical example of their clinical relevance is podophyllotoxin, an aryltetrahydronaphthalene lignan, whose semisynthetic derivatives, etoposide and teniposide, are first-line antineoplastic agents in contemporary cancer chemotherapy regimens (Shah et al., 2021).

Despite their therapeutic importance, a critical and growing gap exists between the global demand for these phytopharmaceuticals and the sustainable supply of their active ingredients. Conventional harvesting of lignans from wild plant populations is unfeasible on an industrial scale due to their low endogenous concentrations and the prolonged ontogenetic development cycles of the producing species (Singh et al., 2024). Furthermore, reliance on wild harvesting places severe anthropogenic pressure on ecosystems, increasing the risk of genetic erosion and biodiversity loss through overexploitation. On the other hand, alternative approaches, such as total chemical synthesis and the optimization of conventional extraction processes, present severe limitations: synthetic routes are often complex and have low stereochemical yields, while large-scale extraction entails high operating costs and an adverse environmental impact due to the intensive use of organic solvents and the generation of xenobiotic byproducts (Tiz et al., 2024). Consequently, plant cell and organ cultures have emerged as scalable alternatives for lignan production that are independent of seasonal and environmental limitations associated with field cultivation (Murata et al., 2015).

To overcome these bottlenecks and ensure the chemical homogeneity essential in the pharmaceutical industry, it is imperative to develop controlled bioproduction platforms complemented by high-resolution analytical tools. In this regard, high-performance liquid chromatography coupled with mass spectrometry (HPLC-MS) and nuclear magnetic resonance (NMR) are essential analytical technologies that allow for unambiguous structural elucidation and ultra-precise quantification of lignan profiles (Yeung et al., 2020; Consonni and Ottolina, 2022).

In this context, *in vitro* plant cell culture is positioned as a robust, scalable, and sustainable biotechnological system. Callus induction through cell reprogramming of explants in media supplemented with plant growth regulators, particularly with a balanced ratio of auxins to cytokinins, allows for the establishment of cell lines with optimized biosynthetic competence (Bulgakov et al., 2018). The transition from these systems to cell suspension cultures facilitates the proliferation of undifferentiated biomass in bioreactors under strictly controlled physicochemical conditions, independent of seasonal or climatic fluctuations (Gabr et al., 2021; Singh et al., 2024). Furthermore, these suspensions can be enhanced through strategies such as clonal selection, metabolic engineering, and biotic or abiotic elicitation to maximize the metabolic flux toward the lignans of interest (Hidalgo et al., 2018). Beyond productive efficiency, this technology ensures the production of reproducible biological matrices that are free of environmental contaminants and aligned with international standards for good manufacturing practices (Smetanska, 2018).

To define the boundaries of this technological approach, this review comprehensively examines the state of the art regarding the biotechnological production of bioactive lignans. The document is structured by first analyzing the fundamental biosynthetic pathways and the main phytochemical sources described in the literature. Subsequently, the limitations of conventional production methods are critically addressed, and bioprocess engineering strategies applied to bioreactor systems are evaluated. A core component of this work is the inclusion of a chemoinformatic analysis of lignans produced using plant tissue cultures. This computational approach represents a crucial methodological contribution, as it enables molecular cataloging, prediction of pharmacokinetic profiles (ADME-Tox), and virtual screening of these metabolites’ affinity for specific therapeutic targets, thereby optimizing the discovery of new phytopharmaceuticals. Finally, the sector’s prospects are discussed, outlining the technical and scientific challenges and the emerging opportunities that will shape this field of study.

## 2 Methodology

The review methodology was structured systematically to ensure transparency and reproducibility in selecting the literature. First, a search strategy was defined using keywords related to plant tissue culture and lignan production, including “lignan biosynthesis”, “callus culture”, “cell suspension culture”, “Suspension Cells lignans”, “hairy roots”, “plant culture scale-up”, “biotechnological production of lignans”, and “biological activities of lignans”.

Searches were conducted in databases recognized for their reliability and multidisciplinary coverage: PubMed/Medline, Web of Science, Scopus (Elsevier), SpringerLink, ScienceDirect (Elsevier), Wiley Online Library, Frontiers Journals, Google Scholar, Taylor & Francis Online, and MDPI Journals. No restrictions were applied based on publication year, including both classic studies and recent literature that provided evidence on the topic.

In the selection phase, 764 articles and reviews were initially identified. Subsequently, inclusion and exclusion criteria were applied. Only English-language publications presenting experimental data or reviews with clear and verifiable information on the production, biosynthesis, extraction, scale-up, or applications of lignans in plant tissue cultures were included. Duplicate articles across databases, those not directly related to the manuscript’s objectives, and those that did not contribute substantial information were excluded.

Finally, the selected studies were analyzed with respect to the type of *in vitro* culture (callus, cell suspensions, transformed roots), the lignan-producing plant species, the specific metabolites reported, bioprocess aspects (yield, scale-up, technical limitations), and applications in pharmaceuticals, nutraceuticals, and cosmeceuticals. This process enabled a structured, critical synthesis of the information, ensuring that the review reflects both the state of the art and future perspectives in lignan biotechnology.

From the selected studies on biotechnological lignan production, 15 lignans were identified and subsequently used to illustrate their distribution across organisms, cell lines, and molecular targets (Section 4). ChEMBL version 34 was used to retrieve bioactivity data for the lignans and neolignans shown in Section 4 via the ChEMBL application programming interface (API). The searches were performed through the activity module using the corresponding molecule_chembl_id identifiers. In the first search, no restrictions were applied to activity type, allowing the compilation of diverse biological activity records, including IC_50_, GI_50_, ED_50_, potency, inhibition, activity, ratio, mortality, among others. This search retrieved 2,296 activity records. After excluding entries lacking a reported target_organism, 1,839 entries were retained for the descriptive analysis. For visualization purposes in Section 5.6, only organisms represented by more than two assays were included. For Section 5.6, target_pref_name entries represented by at least 20 retrieved records were retained. Entries corresponding to *Mus musculus*, *Mythimna separata*, *Plasmodium falciparum*, and tubulin were excluded because they do not represent cell lines. The remaining target_pref_name entries were used to visualize the distribution of lignans across cell lines.

In a second search, the dataset was restricted to assays annotated as binding assays (assay_type = “B”), yielding 387 records. Duplicate entries sharing the same molecule_pref_name and assay_description were removed, resulting in 259 unique records. For visualization purposes, only compounds represented by more than two binding assays were included in the analysis of experimental frequency by natural product. The resulting datasets are available from the corresponding author upon reasonable request.

## 3 Biological and ecological functions of lignans in plant secondary metabolism

Lignans play important roles in plant defense and adaptation. While they share a biosynthetic origin with lignin, lignans have distinct biological roles, serving mainly as biochemical mediators in stress responses and ecological interactions.

Biotic stress, such as pathogen infection or herbivore attack, often induces lignan biosynthesis via the phenylpropanoid pathway. Many lignans exhibit antifungal, antibacterial, and insect-deterrent properties, supporting their role as defense-related metabolites and phytoalexin-like compounds.

Lignans also support abiotic stress tolerance. Their antioxidant activity reduces reactive oxygen species produced during drought and extreme temperatures, thereby sustaining metabolic function.

Beyond stress responses, lignans accumulate in reproductive and storage tissues such as seeds and roots, indicating roles in plant development and reproductive fitness. They may also influence ecological interactions, including allelopathy, and are linked to the regulation of lignification and cell wall metabolism (Ražná et al., 2021; Ninkuu et al., 2022; Bagniewska-Zadworna et al., 2014; Zhang et al., 2014; Singh et al., 2024).

Overall, evidence shows that lignans act as multifunctional metabolites, integrating defense, oxidative stress protection, and ecological adaptation. These roles are closely tied to the activation of the lignan biosynthetic pathway, which supports many elicitation and metabolic engineering strategies discussed later in this review.

## 4 Biosynthesis and chemodiversity of lignans

Lignans are a family of phenolic compounds widely distributed in vascular plants. Structurally, they consist of two phenylpropanoid (C6-C3) units derived from the amino acids *L*-phenylalanine and *L*-tyrosine. Their biogenesis originates in the shikimic acid pathway, where the enzyme phenylalanine ammonia-lyase (PAL) catalyzes the first step, the deamination of phenylalanine, to generate cinnamic acid (Weng and Chapple, 2010). Through a series of hydroxylation and methylation reactions, this intermediate is transformed into monolignol alcohols, with coniferyl alcohol being the central precursor of most lignans (Petersen et al., 2010).

The process continues with the stereoselective oxidative dimerization of two coniferyl alcohol units (**Figure 1**). This reaction is mediated by peroxidases or laccases and directed by dirigent proteins (DIRs), which precisely control the orientation and chirality of the phenolic coupling (Markulin et al., 2019). This interaction leads to the formation of (+)- or (-)-pinoresinol, thus establishing the absolute configuration of the metabolite. Subsequently, pinoresinol is sequentially transformed into lariciresinol and secoisolariciresinol by the action of NADPH-dependent reductases (such as pinoresinol-lariciresinol reductase, PLR). Secoisolariciresinol acts as the structural scaffold from which the numerous lignan families of therapeutic interest are derived. Among the plant genera studied for lignan production, *Linum* is one of the most extensively investigated, particularly for its biotechnological potential (Deng & Lu, 2017; Saadullah, 2025; Dewick, 2009).

**Figure 1:**
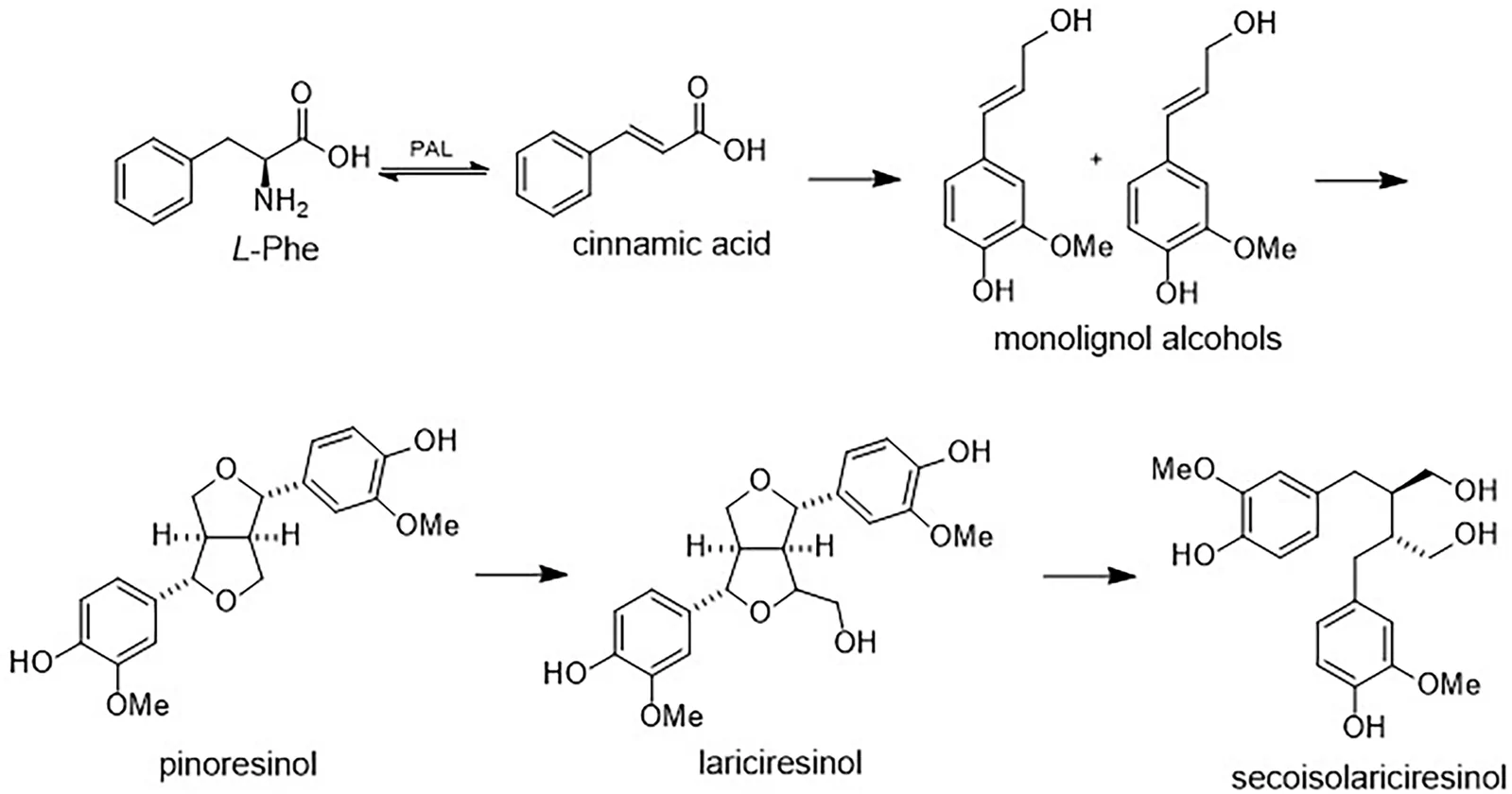
General biosynthetic pathway of lignans.

Lignan chemodiversity results from different cyclization patterns that occur after the oxidative coupling of two phenylpropane units (C_6_–C_3_–C_6_–C_3_), a process that occurs predominantly at the 8 and 8′ positions (Plaha et al., 2022; Ushimaru, 2024). Variations in side-chain oxidation and ring formation generate the structural diversity that underlies the classification of lignans into several major subgroups (Satake et al., 2015; Ražná et al., 2022; Moss, 2000), such as dibenzylbutane, dibenzylbutyrolactone, furan, furofuran, and aryltetralin lignans, as well as neolignans. According to their structural classification, certain lignan types are predominantly associated with specific plant genera. Among these, *Linum* stands out as the most representative genus, as it has been reported to biosynthesize nearly all major lignan classes (**Table 1**).

**Table 1:** Structural classification of lignans and their most representative plant genera.

Lignan type	General structure	Example of representative gender	Reference
Lignans			
Dibenzylbutane	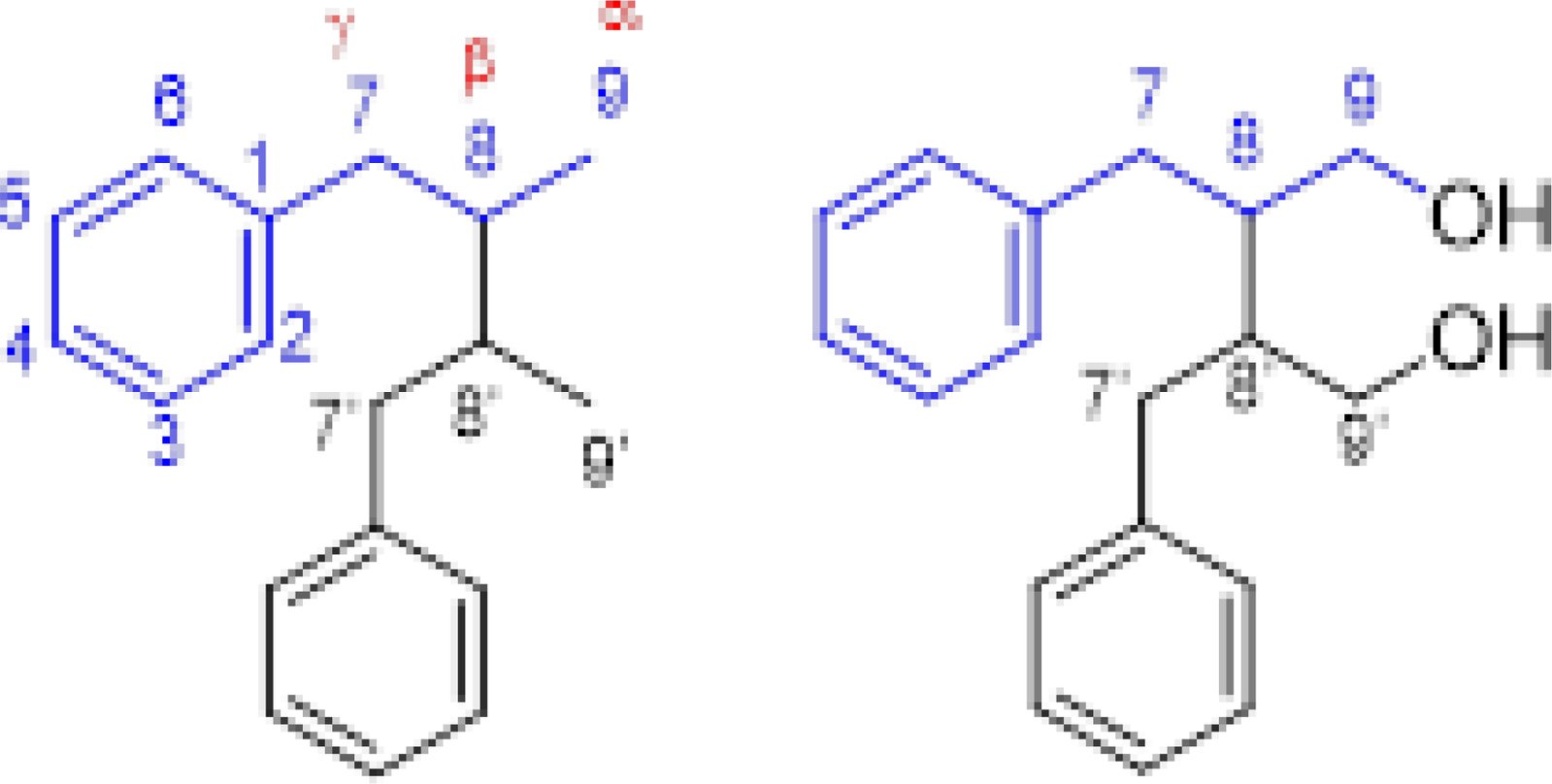	*Linum*, *Forsythia*	(Kezimana et al., 2018)
Dibenzylbutyrolactone	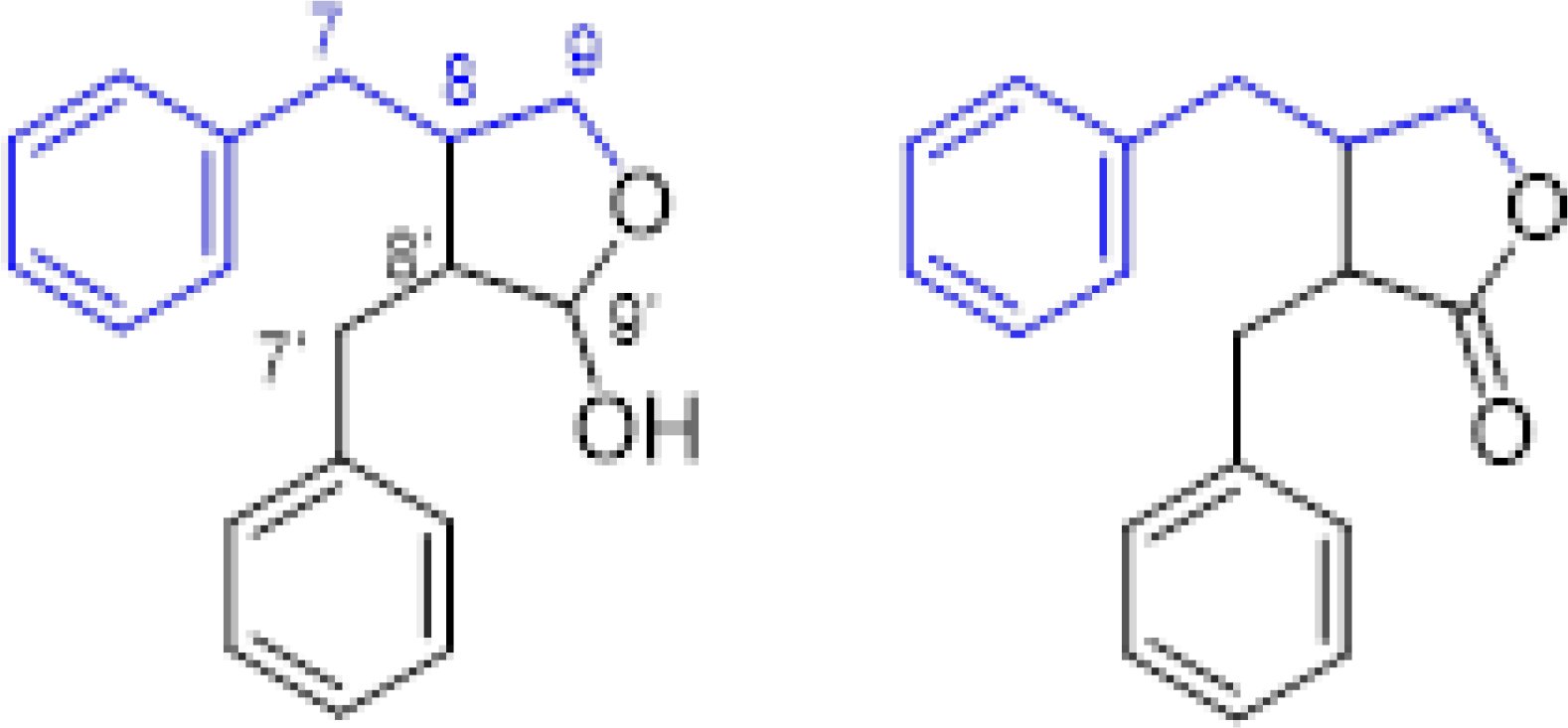	*Forsythia*, *Arctium*	(Rahman et al., 1990; Wu et al., 2022)
Furofuran	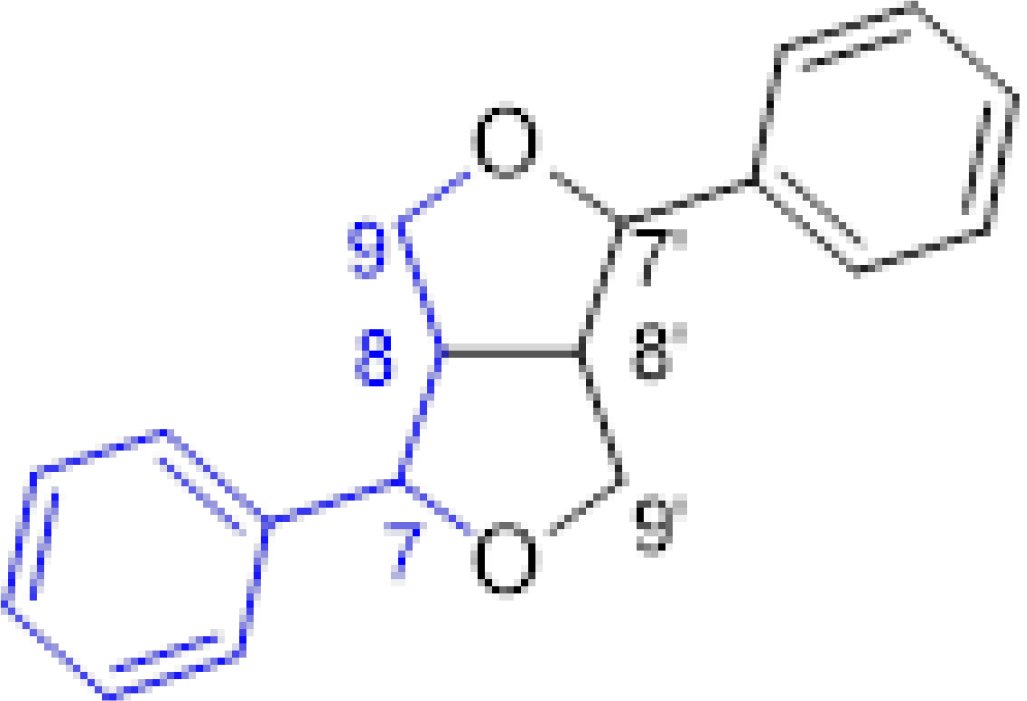	*Sesamum*, *Forsythia*	(Rahman et al., 1990; Andargie et al., 2021)
Furan	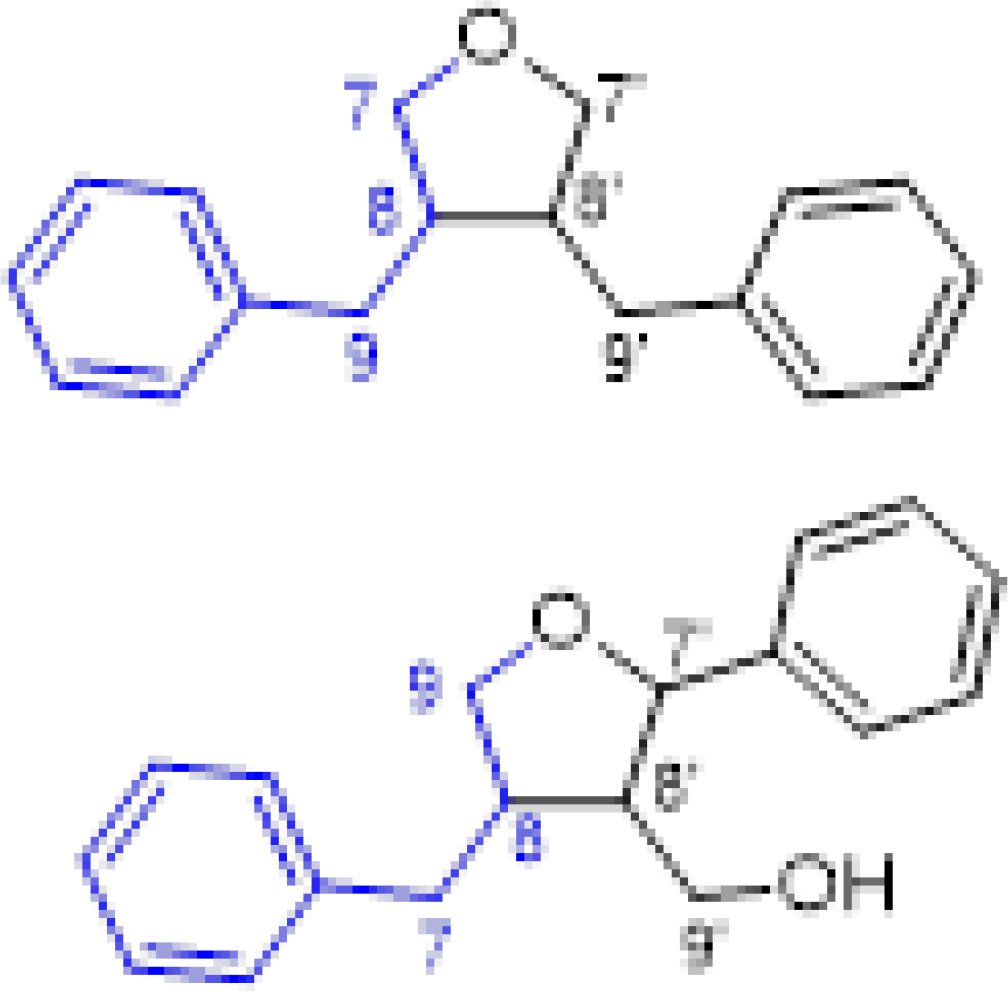	*Linum*, *Brassica*	(Hemmati et al., 2010)
Ciclolignans			
Ariltetralin	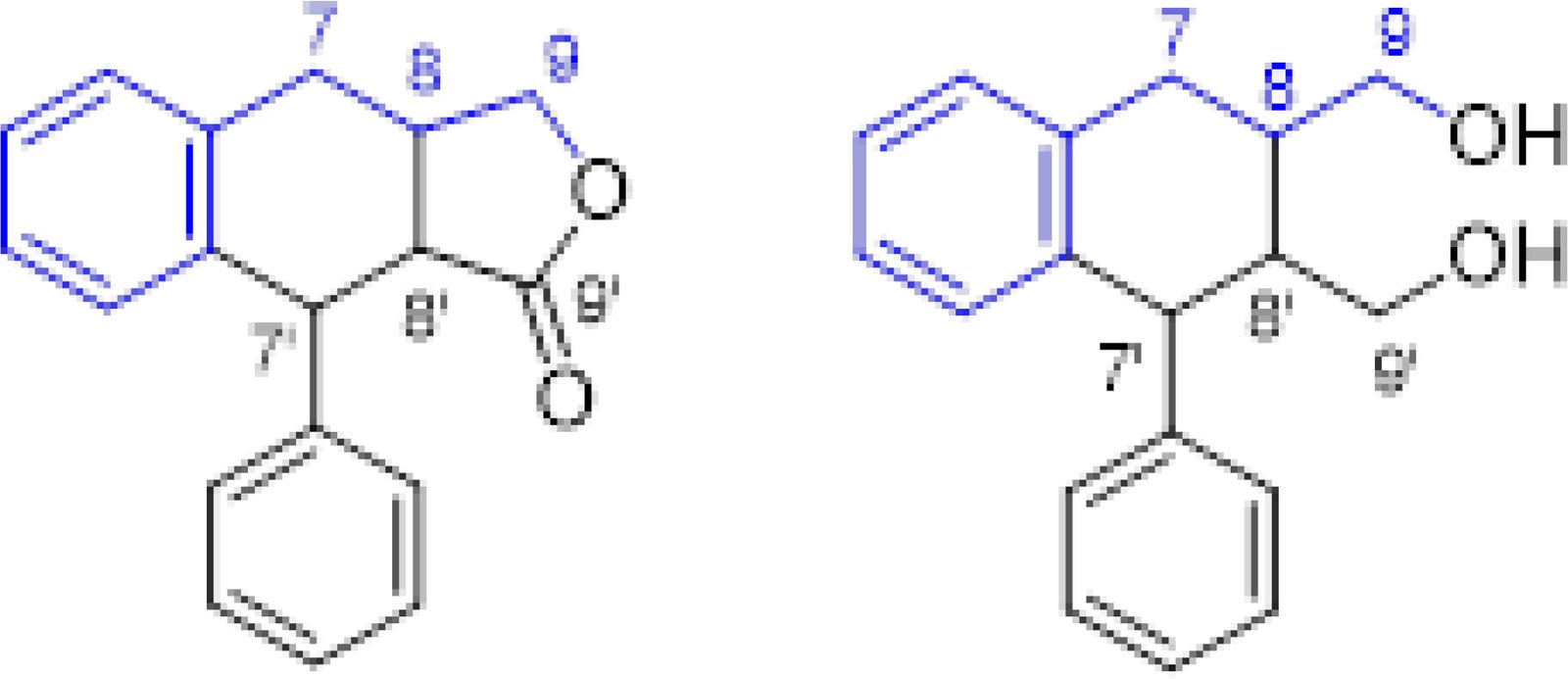	*Podophyllum*, *Linum*	(Schmidt et al., 2010; Kumar et al., 2022)
Arilnaphtalene	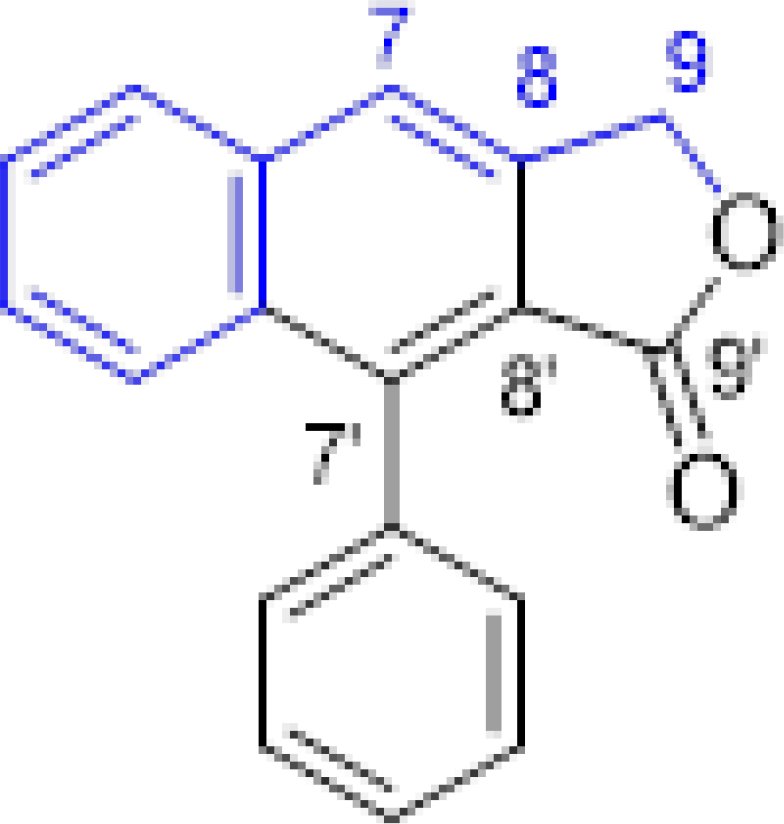	*Justicia*, *Linum*	(Hemmati and Seradj, 2016; Nedelcheva et al., 2024)
Dibenzocyclooctadiene	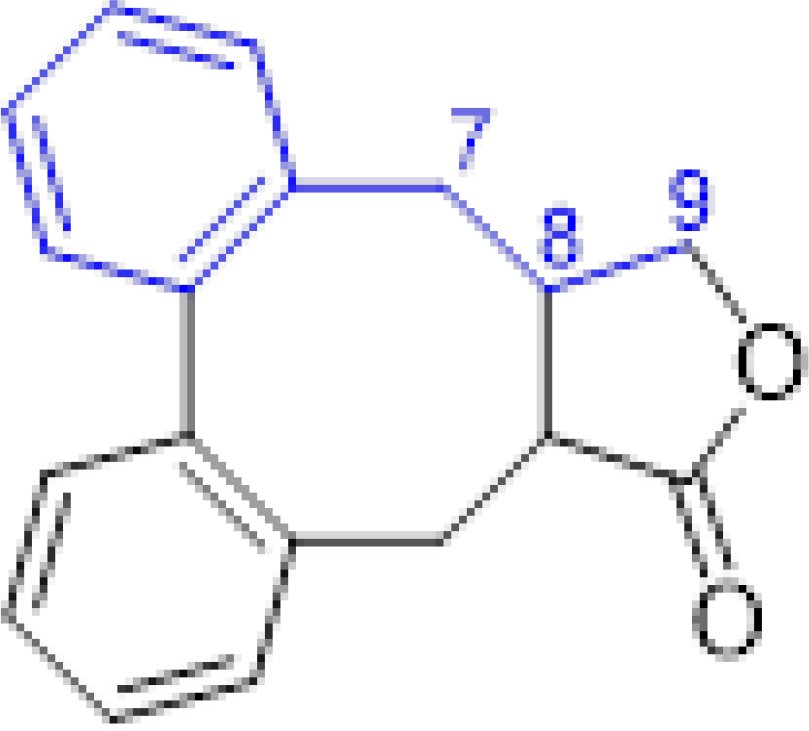	*Schisandra*	(Liu et al., 2023)
Neolignans			
3-3’ neolignans 8-3’ neolignans	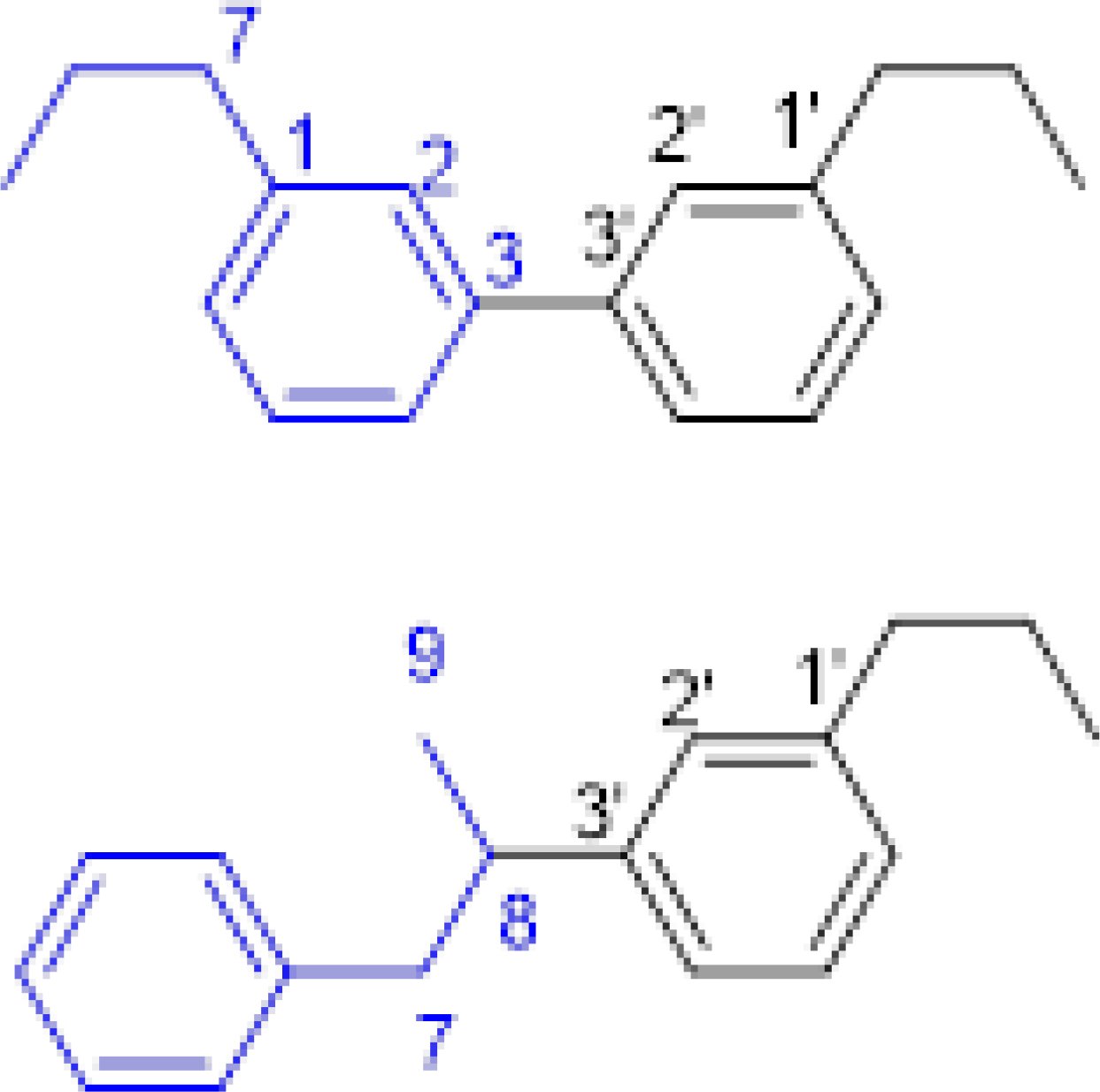	*Magnolia*	(Li et al., 2024)

The simplest lignans correspond to the dibenzylbutanes, characterized by a linear chain of four carbon atoms connecting both aromatic rings. From this basic skeleton, intramolecular oxidation and incorporation of a lactone motif into the side chain yield the dibenzylbutyrolactones, a subgroup of great biosynthetic relevance that includes key intermediates such as matairesinol (Plaha et al., 2022). Furthermore, the formation of intramolecular ether linkages favors the emergence of tetrahydrofuran-type heterocyclic structures, giving rise to two distinct families: furans, which contain a single heterocyclic ring, and furofurans, characterized by a fused bicyclic system with high rigidity and conformational stability, classically exemplified by (+)-pinoresinol and sesamin (Weng and Chapple, 2010).

A higher level of structural complexity is observed in cyclolignans, particularly in aryltetralins, where an additional intramolecular cyclization between one of the aromatic rings and the side chain results in a fused tetralin system. Podophyllotoxin, a prominent representative of this group, occupies a central place in modern oncology, both for its antimitotic activity and for its role as a precursor to widely used semisynthetic chemotherapeutic agents. Closely related are the arylnaphthalene lignans, distinguished by the complete aromatization of the central naphthalene system (Dewick, 2009). Furthermore, in certain botanical families, such as the Schisandraceae, dibenzocyclooctadienes have been identified (Jafernik et al., 2024b; Rybnikář et al., 2024). These are particularly unique structures that feature a central eight-membered ring. This structure results from additional oxidative coupling between aromatic rings and represents one of the most structurally complex lignan subclasses.

It is essential to distinguish these compounds from neolignans, whose molecular architecture does not conform to the classic 8–8′ linkage pattern, but rather involves alternative bonds such as 8–3′, 3–3′, or 8–O–4′. This structural divergence significantly expands the accessible chemical space within this metabolite family, generating molecular arrangements with distinct physicochemical properties and biological activities (Plaha et al., 2022; Gao et al., 2024). Finally, the classification of lignans is completed with extreme structural variants. These include norlignans, characterized by the loss of a carbon atom in the side chain (C_17_), and oligolignans, resulting from the condensation of three or more phenylpropane units, which give rise to sesquilignans and dilignans of high molecular complexity (Boerjan et al., 2003; Weng and Chapple, 2010).

Based on the selected studies on lignan production in plant tissue cultures, fifteen lignans and neolignans were identified. Their structural classification is presented in **Figure 2**, while information on the plant species of origin, culture platforms, and production yields is summarized in Section 7.4.1 and Section 7.4.4. These fifteen compounds were subsequently used for the bioactivity analysis presented in Section 5.6 and **Figure 3**.

**Figure 2:**
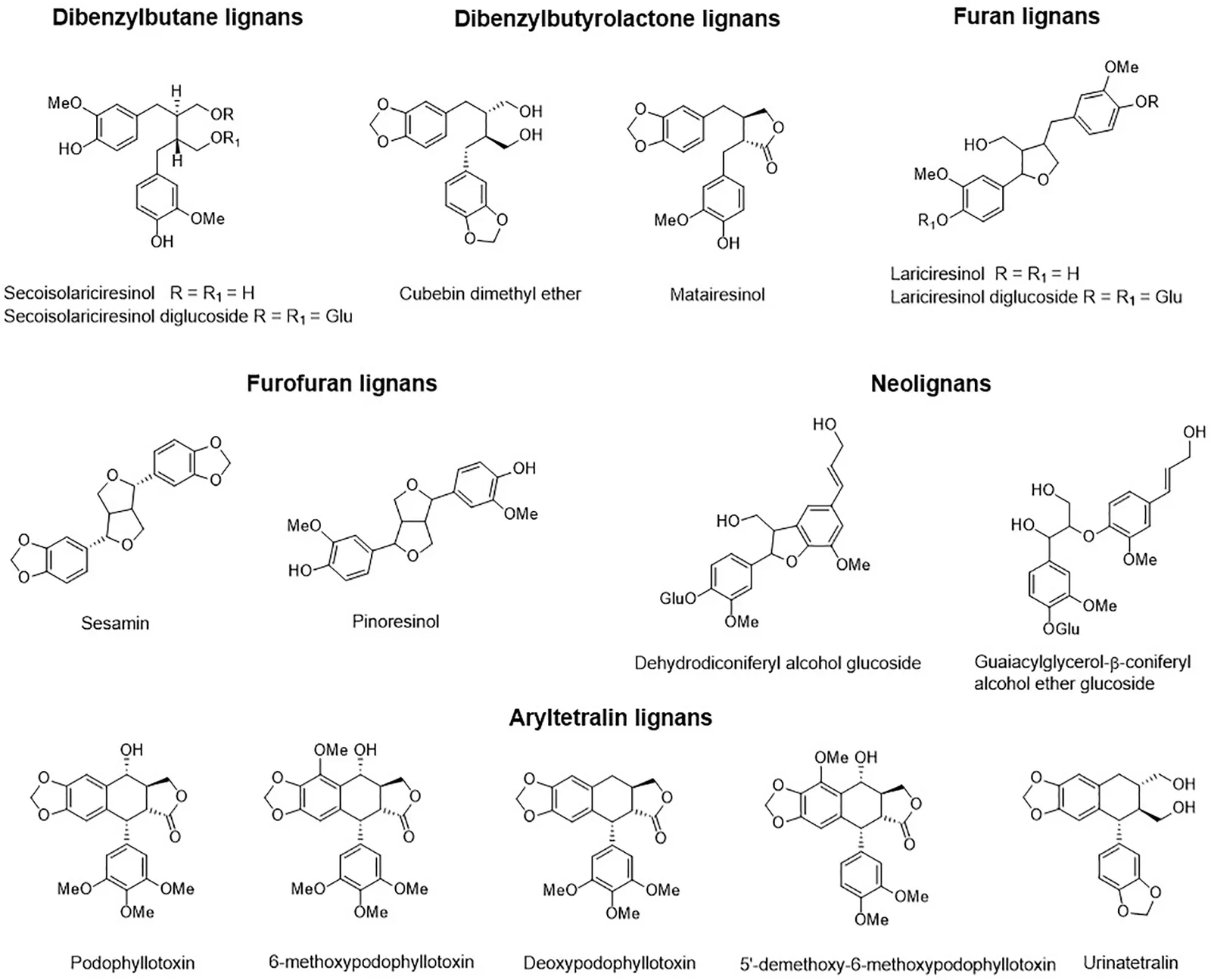
Lignans and neolignans obtained by biotechnological methods.

**Figure 3:**
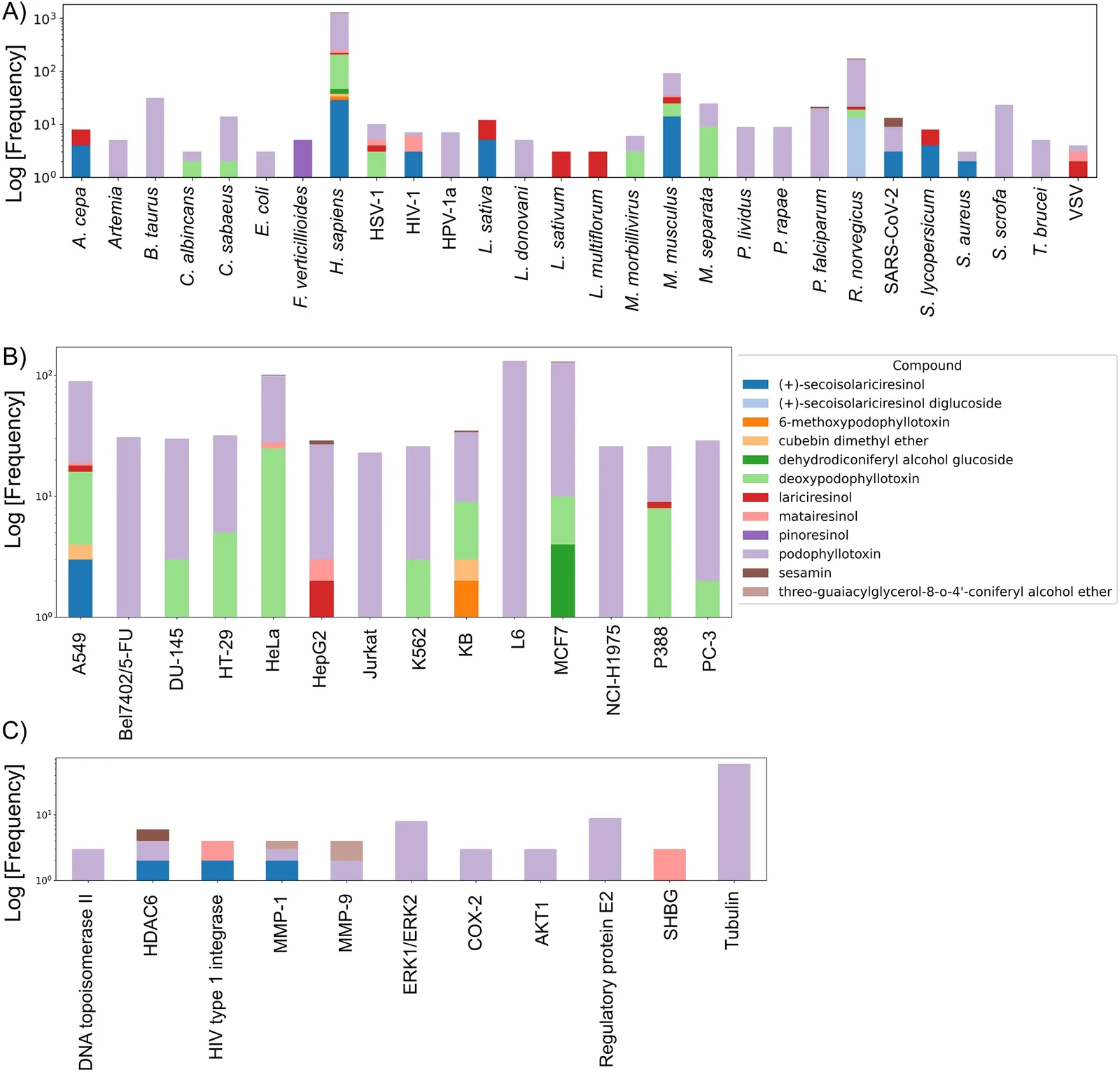
Representation of lignans and neolignans across reported **(A)** organisms, **(B)** cell lines, and **(C)** biological targets. The y-axis represents the logarithm of the number of reported assay entries. Stacked colored segments indicate the contribution of each lignan or neolignan to the total number of assay entries within each organism, cell line, or biological target category.

## 5 Overview of pharmacological applications of lignans

### 5.1 Lignans as antineoplastic agents

Lignans have been widely studied in experimental oncology due to their structural diversity and ability to interact with various molecular targets. Podophyllotoxin, isolated from *Podophyllum peltatum* and *P. emodi*, is among the most extensively researched lignans because of its strong cytotoxic effects. This compound functions as an antimitotic agent by disrupting microtubule dynamics and causing cell-cycle arrest at the M phase. Despite its potency, clinical use of podophyllotoxin is restricted by significant systemic toxicity and a narrow therapeutic index, prompting the development of semisynthetic derivatives with enhanced therapeutic profiles (Ardalani et al., 2017; Fan et al., 2021).

This approach led to the development of etoposide and teniposide, two highly successful lignan-derived anticancer agents (Cheng et al., 2014; Shah et al., 2021). In contrast to podophyllotoxin, these drugs primarily inhibit topoisomerase II, resulting in DNA damage and apoptosis in rapidly dividing cells. Both etoposide and teniposide remain essential in the clinical management of small-cell lung cancer, testicular cancer, leukemias, and lymphomas (Sun et al., 2022; Motyka et al., 2023).

Several other lignans are in earlier stages of development. Nordihydroguaiaretic acid (NDGA) has been shown to inhibit both 5-LOX and IGF-1R, thereby reducing tumor proliferation in breast and prostate adenocarcinoma models (Lambert et al., 2004; Mala John et al., 2020). Honokiol is notable for its ability to cross the blood-brain barrier and inhibit the mTOR pathway in glioblastoma models (Ong et al., 2019). In comparison, arctigenin and sauchinone have demonstrated antiproliferative effects primarily *in vitro*, including activity against pancreatic cancer cells and modulation of pathways related to epithelial-mesenchymal transition (He et al., 2018; Shabgah et al., 2021).

Collectively, current evidence indicates that lignan-derived antineoplastic agents are at varying stages of development, ranging from clinically approved drugs such as etoposide and teniposide to promising preclinical candidates that warrant further investigation.

### 5.2 Antioxidant lignans and neuroprotection: molecular mechanisms and challenges

Oxidative stress is a central factor in the pathophysiology of neurodegenerative diseases, thereby driving research into lignans such as sesamin and honokiol (Li et al., 2023). Permeability to the central nervous system is critical: honokiol possesses physicochemical characteristics that favor its passive crossing of the blood-brain barrier, although it still requires *in vivo* validation (Wan et al., 2015; Arab et al., 2022).

*In vitro* studies show that these lignans neutralize reactive oxygen species and prevent lipid peroxidation, thereby preserving neuronal integrity (Polat Kose and Gulcin, 2021; Olas, 2025). Furthermore, they promote the nuclear translocation of Nrf2, which activates antioxidant genes such as glutathione peroxidase, superoxide dismutase, and catalase (Lianlin et al., 2019; He et al., 2023; Bernatoniene et al., 2026). Although preclinical data suggest neuroprotective benefits, clinical efficacy, optimal dosage, and long-term safety in pathologies such as Alzheimer’s and Parkinson’s diseases have not yet been validated. Therefore, sesamin and honokiol should be considered experimental chemical scaffolds for the design of future therapeutic agents.

### 5.3 Lignans as phytoestrogens and immunomodulators

The phytoestrogenic activity of lignans depends on their biotransformation by the gut microbiota, which converts dietary compounds such as SDG, matairesinol, pinoresinol, and lariciresinol into active enterolignans, primarily enterodiol and enterolactone (Lampe et al., 2006; Carreau et al., 2008). This conversion varies among individuals, leading to differences in circulating concentrations (Li et al., 2022). Enterolactone acts as a weak ligand for ERα and ERβ, regulating gene transcription and selectively modulating estrogen receptors (Carreau et al., 2008; Jang et al., 2022). It also influences nuclear pathways such as PPAR and LXR, thus broadening its metabolic impact (Drygalski et al., 2017; Laveriano-Santos et al., 2025).

SDG has shown anti-inflammatory effects by inhibiting the NF-κB pathway and regulating the NLRP3 inflammasome in *in vivo* models (Zhang et al., 2020; Zhong et al., 2026). Sesame-derived lignans, such as sesamin and sesamolin, reduce pro-inflammatory mediator levels and promote IL-10 production (Zhong et al., 2026). Arctigenin has been associated with the regulation of innate and adaptive immune responses. Although some epidemiological studies link elevated enterolactone levels with a lower risk of breast cancer (Saarinen et al., 2007; Tułacz and Włodarczyk, 2026), the evidence is inconsistent and depends on genetic, hormonal, and dietary factors (Zanella et al., 2017). Furthermore, phytoestrogenic activity carries potential contraindications in sensitive populations and risk of drug interactions (Lampe et al., 2006; Sun et al., 2022).

### 5.4 Lignans as anti-inflammatory and antiviral agents

Honokiol and arctigenin inhibit NF-κB activation and nuclear translocation, thereby reducing TNF-α and IL-6 expression and attenuating inflammation (Gao et al., 2018). NDGA acts as a dual inhibitor of COX-2 and 5-LOX, modulating the biosynthesis of pro-inflammatory eicosanoids (Mala John et al., 2020; Manda et al., 2020).

In the antiviral domain, sauchinone inhibits viral enzymes such as RNA polymerase and reverse transcriptase (Saadh et al., 2021). Podophyllotoxin derivatives have shown activity against influenza and SARS-CoV-2 by disrupting viral assembly and intracellular replication (Jin et al., 2022; Strus et al., 2025). Arctigenin combines anti-inflammatory and antiviral effects, interfering with the entry, assembly, and release of infectious particles in influenza, chikungunya, and SARS-CoV-2 models (Hayashi et al., 2010; Shukla et al., 2024).

Although experimental results are promising, most are from *in vitro* and *in vivo* studies, so clinical efficacy still requires validation.

### 5.5 Non-therapeutic applications of lignans

In addition to their biomedical effects, lignans have garnered increasing interest for their potential in non-therapeutic applications, encompassing the food industry, sustainable agriculture, cosmetics, and the development of bio-based materials (Soleymani et al., 2020; Vasile and Baican, 2023; Berenshtein et al., 2024). This versatility is based on their redox properties, antimicrobial activities, and structural characteristics, positioning them as candidates within the paradigms of circular bioeconomy and green chemistry to replace petrochemical inputs with renewable compounds (Mizani et al., 2025).

In the food sector, *in vitro* studies have shown that lignans such as sesamin and sesamolin inhibit lipid peroxidation in vegetable oils and meat matrices, even under conditions of thermal processing or prolonged storage (Teshome et al., 2022). These results suggest their potential as high-performance natural antioxidants. However, the magnitude of their effect depends on the food matrix and processing conditions, so systematic characterization is still needed to establish efficacy parameters comparable to traditional synthetic antioxidants (BHA, BHT).

In the agricultural sector it has been demonstrated that lignans possess antifungal, antibacterial, and insecticidal activity (Kyselka et al., 2017). These findings position them as promising molecules for the development of selective biopesticides, with potential advantages in terms of biodegradability and lower environmental toxicity compared to synthetic agrochemicals. Furthermore, certain podophyllotoxin derivatives have shown allelopathic effects, modulating the germination and growth of competing species (Kato-Noguchi et al., 2023; Yamauchi, 2024; Li et al., 2025). However, most of these results come from controlled laboratory or greenhouse studies, so their applicability in extensive agricultural systems still requires validation under field conditions.

In the cosmetic and personal care sector, standardized extracts of flax (*L. usitatissimum*), sesame (*S. indicum*), and magnolia (*Magnolia* spp.) are currently being incorporated into cosmeceutical formulations. Their use is based on experimental evidence supporting their antioxidant and photoprotective capacity against oxidative stress induced by ultraviolet radiation, as well as their function as emulsion stabilizers and skin barrier protectors (Neelam et al., 2020; Sangiorgio et al., 2023). In this case, the application is supported by dermatological safety data and regulatory approval, which have allowed their inclusion in consumer products available on the market.

Overall, lignans are emerging as a promising class of natural compounds with broad potential in non-therapeutic applications. Their versatility is evident across various sectors, though to varying degrees. In the food industry, laboratory studies have demonstrated their antioxidant and antimicrobial capacity, suggesting a significant role as natural stabilizers against oxidative deterioration. In agriculture, experimental results indicate their usefulness as biopesticides and allelopathic agents, with implications for sustainable and ecological practices. However, these findings still need to be translated into field settings to confirm their efficacy. In contrast, the cosmetics sector has already incorporated flax, sesame, and magnolia extracts into commercial formulations, with evidence of dermatological safety and regulatory approval, thereby consolidating their practical application.

Therefore, lignans represent a bridge between basic research and applied innovation. While some uses remain in exploratory phases and require further validation, others have reached a sufficient level of maturity to be integrated into consumer products. This distinction underscores the importance of evaluating each application according to its level of evidence, avoiding premature extrapolations and recognizing both its possibilities and its current limitations.

### 5.6 Overview of reported bioactivities of biotechnologically produced lignans and neolignans

To provide a descriptive overview of the reported biological activities of lignans and neolignans obtained by biotechnological methods (**Figure 2**), the associated organisms, cells, and biological targets were analyzed. Bioactivity data shown in **Figure 3** were retrieved from the ChEMBL 34 database using the application programming interface (API) (Zdrazil et al., 2024). This search retrieved 2,296 activity records with heterogeneous assay types. A second search was restricted to assays annotated with the assay type “B”, corresponding to binding assays associated with specific biological targets, yielding 387 unique entries after duplicate removal.

This approach enabled the compilation of bioactivity information reported in the literature through three complementary strategies: a general overview of reported biological activities and cell lines, and a target-focused search restricted to affinity (binding) assays. The global bioactivity dataset comprised heterogeneous pharmacological parameters, including IC_50_, GI_50_, and EC_50_ values. Due to the diversity of assay endpoints, the data were interpreted descriptively, and no direct quantitative comparisons between assay types were performed. **Figure 3** summarizes the reported activities across organisms, cell lines, and biological targets. A total of 1,839 unique assays associated with the compounds shown in **Figure 3A** with available target_pref_name were included in the descriptive analysis.

#### 5.6.1 Bioactivity assays across organism and species

The representation in **Figure 3A** reflects the available records of reported bioactivity assays across organisms and species and does not indicate the natural occurrence or distribution of lignans and neolignans. As illustrated in panel A, research efforts are heavily biased toward *Homo sapiens* (1,290 assays), highlighting the potential of lignans in human therapeutics. Within this area, extensive evaluations have mainly focused on tumor cell lines such as MCF-7, HeLa, A549, KB, and HT-29, in which (+)-secoisolariciresinol, podophyllotoxin, and deoxypodophyllotoxin emerged as the most representative compounds. The latter two compounds were primarily associated with cancer-related assays. *Rattus norvegicus* L6 cells were mainly evaluated with podophyllotoxin, deoxypodophyllotoxin, and lariciresinol, whereas *Mus musculus* P388 cells were predominantly tested with (+)-secoisolariciresinol, deoxypodophyllotoxin, and podophyllotoxin, with fewer assays involving lariciresinol. The recurrent use of podophyllotoxin in cytotoxicity and tubulin-polymerization assays, including studies involving resistant cancer cell lines, highlights its continued relevance as a pharmacological reference compound in anticancer drug design. Moreover, despite the development of podophyllotoxin derivatives with comparable antiproliferative activity, podophyllotoxin itself remains a prevalent benchmark for evaluating potency and resistance-associated phenotypes in tumor models (Romagnoli et al., 2006; Hyder et al., 2015; Wang et al., 2018b).

#### 5.6.2 Bioactivity assays across cell lines and molecular targets

The bioactivity assay data depicted in **Figure 3B** and C reveal that podophyllotoxin remains the most extensively studied natural product among the compounds analyzed. In addition, biological evaluations beyond podophyllotoxin-related anticancer activity have also been reported. For example, sesamin and podophyllotoxin exhibit activity against HDAC6 (histone deacetylase 6) and MMP-9 (matrix metalloproteinase 9), suggesting potential applications in neurodegenerative disorders, chronic inflammatory diseases, and cancer malignancies (Dick et al., 2021; Qiu et al., 2021). For example, the regulatory proteins E2 and E7 of human papillomavirus type 16 (HPV16) act as transcriptional regulators and DNA replication regulators and are associated with cancer. In this context, podophyllotoxin has been described as a weak inhibitor of the protein–protein interaction between E2 and E7 (Saitoh et al., 2008). Other lignans have also been evaluated against cancer-associated proteolytic enzymes. In this context, secoisolariciresinol showed marginal inhibition of matrix metalloproteinase-1 (MMP-1) (IC_50_ = 50 μM), whereas guaiacylglycerol coniferyl alcohol ether exhibited improved activity against MMP-9, with an IC_50_ value of 9.9 μM (Wang et al., 2012). Furthermore, a 2-acetoxybenzoate derivative of podophyllotoxin suppressed the phosphorylation of RAC-alpha serine/threonine-protein kinase (AKT1), a signaling pathway associated with tumor progression, metastasis, and drug resistance (Zhang et al., 2017; Wang et al., 2018b). Modulation of the extracellular signal-regulated kinase 1/2 (ERK1/2) pathway has also been reported for podophyllotoxin derivatives. In Bel-7402/5-FU cells, podophyllotoxin inhibited the phosphorylation of ERK1/2 and AKT, suppressing cell migration, whereas conjugates derived from podophyllotoxin and non-steroidal anti-inflammatory drugs produced distinct regulatory effects on ERK1/2, AKT, and STAT3 signaling pathways (Zhang et al., 2017). Collectively, these findings suggest that structural modifications of podophyllotoxin can differentially modulate signaling pathways involved in multidrug resistance, proliferation, and metastasis, highlighting their potential as antineoplastic agents.

On the other side, podophyllotoxin did not exhibit activity against cyclooxygenase-2 (COX-2) (Huss et al., 2002), whereas deoxypodophyllotoxin showed inhibitory concentrations below 2 μM, displaying selective COX-2 inhibition and activity comparable to indomethacin (Lee et al., 2004; Guerrero et al., 2013).

The lignans (+)-secoisolariciresinol and matairesinol did not inhibit human immunodeficiency virus type 1 (HIV-1) integrase; however, one of their analogs strongly inhibited both 3′-processing and strand transfer, with inhibition levels of 92% and 100%, respectively. Additional details regarding the assay and compound structure can be found in the original report (Eich et al., 1996). Additional antiviral activities have been reported for deoxypodophyllotoxin, podophyllotoxin, lariciresinol, and matairesinol against several viral targets. Activity against herpes simplex virus type 1 (HSV-1) was evaluated in CV-1 cells, where podophyllotoxin exhibited an IC_50_ of 0.05 μM, while deoxypodophyllotoxin was approximately two-fold more potent. In Vero cells, podophyllotoxin showed 11.7% inhibition of HSV-1 replication. Anti-HIV-1 activity was assessed in H9 cells, in which matairesinol exhibited an EC_50_ of 2 μM, corresponding to 50% inhibition of viral replication. In addition, podophyllotoxin demonstrated activity against vesicular stomatitis virus (VSV) in BHK cells, with an IC_50_ value of 0.1 μM (Charlton, 1998; Ishida et al., 2001). However, these antiviral activities were not included in the target-based analysis shown in **Figure 3** because no specific molecular targets were reported in the corresponding studies.

Finally, the enantiomers (−)-secoisolariciresinol (IC_50_ = 230 μM) and (−)-matairesinol (IC_50_ = 56 μM) inhibit the binding of dihydrotestosterone to sex hormone–binding globulin (SHBG), a transport protein that, in the presence of dihydrotestosterone, promotes the growth of prostate cancer ALVA-41 cells (Schöttner et al., 1998). This analysis of reported bioactivities not only validates the historical use of certain lignans but also identifies knowledge gaps in which exploring less common derivatives could lead to new chemical entities with high selectivity.

## 6 Pharmaceutical formulations of lignans

The polarity of lignans varies considerably depending on their degree of glycosylation and specific structural features. Glycosylated lignans are typically more polar and water-soluble, whereas aglycones and enterolignans display greater lipophilicity. This variation directly influences their distribution and interaction with biological systems (Wang et al., 2022).

Aglycone lignans generally exhibit moderate partition coefficients (log P), favoring membrane permeability and facilitating their transport across biological barriers. Moreover, the phenolic hydroxyl groups in these compounds typically have pKa values between 8 and 10, indicating that lignans predominantly remain in their non-ionized form under physiological conditions, thereby further enhancing passive diffusion (Devi, 2019). Lignans’ physicochemical behavior is influenced by their capacity to form hydrogen bonds and engage in hydrophobic interactions. These properties affect their affinity for biological membranes and plasma proteins, tissue distribution, and overall pharmacokinetics (Pilkington, 2018).

Despite their favorable physicochemical characteristics and therapeutic potential, the pharmaceutical development of many lignans is limited by poor aqueous solubility, chemical instability, susceptibility to oxidative degradation, extensive first-pass metabolism, and the resulting low oral bioavailability. These limitations have driven the development of advanced formulation strategies, including phytosomes, liposomes, nanoemulsions, polymeric nanoparticles, and semisynthetic derivatives, to improve their physicochemical properties, enhance bioavailability, and optimize their pharmacokinetic performance (Devi, 2019).

Because the pharmaceutical performance of lignans is strongly influenced by their physicochemical properties, their pharmacokinetic characteristics, including absorption, distribution, metabolism, excretion, and toxicity (ADMET), have been extensively investigated to evaluate their suitability as drug candidates (Torres-Moreno et al., 2024). One of the most widely used approaches for this purpose is assessing drug-likeness using Lipinski’s Rule of Five, which identifies compounds with physicochemical properties generally associated with favorable oral bioavailability, including appropriate molecular weight, lipophilicity, and solubility (Lipinski et al., 1997). Compounds that satisfy these criteria are generally considered more likely to exhibit adequate absorption and pharmacokinetic performance. Nevertheless, the pharmacokinetic behavior of lignans is influenced by multiple physiological factors beyond their intrinsic physicochemical properties. Many lignans exhibit limited oral bioavailability owing to poor aqueous solubility, intestinal metabolism, and extensive first-pass metabolism. In addition, glycosylated lignans require hydrolysis by the intestinal microbiota before absorption, and the resulting enterolignans undergo extensive phase II metabolism, contributing to considerable interindividual variability in systemic exposure. Consequently, favorable drug-like characteristics alone are insufficient to ensure optimal therapeutic performance. These pharmacokinetic limitations highlight the importance of developing pharmaceutical formulation strategies to improve solubility, stability, absorption, and overall therapeutic efficacy (Mueed et al., 2024).

Second, the lead-like profile comprises structurally simpler compounds with lower molecular weight and reduced lipophilicity (LogP), making them suitable starting points for chemical optimization to improve pharmacological and pharmacokinetic properties. Finally, the known drug space (KDS) encompasses compounds that have been evaluated in humans and are associated with clinically relevant chemical space (Oprea et al., 2001). These classification frameworks provide a useful basis for evaluating the pharmaceutical potential of lignans and for identifying compounds with favorable characteristics for further drug development. Among lignans, podophyllotoxin represents one of the best-known examples of successful pharmaceutical development, illustrating the potential of this class of compounds as scaffolds for drug discovery. Further advances derived from podophyllotoxin-based semisynthetic derivatives are discussed in the following section on pharmaceutical formulations.

In a chemometric study conducted by Pilkington (2018), 160 lignans and structurally related compounds were evaluated to characterize their physicochemical properties and assess their distribution within established pharmaceutical chemical spaces. Although none of the compounds met all criteria for a lead-like profile, more than 75% were classified within the drug-like space, indicating physicochemical characteristics generally considered favorable for pharmaceutical development (Pilkington, 2018). Additionally, approximately 97.5% of the evaluated compounds were located within the known drug space (KDS), reflecting their structural similarity to compounds associated with clinically relevant chemical space. These findings suggest that many lignans possess physicochemical attributes compatible with pharmaceutical development, although their successful therapeutic application still depends on overcoming important biopharmaceutical limitations through appropriate formulation strategies. Within the dataset analyzed by Pilkington (2018), several lignan subclasses possessed physicochemical properties compatible with pharmaceutical development, supporting their continued investigation as potential drug candidates. Furthermore, the chemometric analysis suggested that lignans generally displayed more favorable drug-like profiles than flavonolignans and other related compounds (CLCs). This difference was primarily attributed to the higher polarity and greater structural flexibility of flavonolignans, which more frequently fell outside the drug-like chemical space. These observations should be interpreted as findings from this specific chemometric study and warrant further validation through additional analyses of larger, more diverse lignan datasets.

Although the absorption of lignans varies according to their chemical structure, several studies have reported that dibenzocyclooctadiene lignans isolated from *Schisandra chinensis*, particularly their glycosylated derivatives, exhibit limited direct gastrointestinal absorption (Adlercreutz, 2007).

Following hydrolysis by intestinal enzymes and microbial β-glucosidases in the gut microbiota, glycosylated lignans are converted into aglycones that can subsequently be metabolized into enterolignans such as enterodiol and enterolactone (Plaha et al., 2022). Following absorption, lignans and their metabolites are distributed throughout the body according to their physicochemical properties and degree of conjugation, with preferential accumulation reported in organs such as the liver and lungs. Their presence has also been detected in the central nervous system, suggesting that certain lignans can cross the blood–brain barrier (Torres-Moreno et al., 2024). The extent of systemic exposure may vary considerably among individuals due to differences in gut microbiota composition, metabolic activity, and physiological status, highlighting the importance of formulation strategies to improve pharmacokinetic performance and therapeutic outcomes.

Lignan metabolism occurs predominantly in the liver, where cytochrome P450 enzymes mediate reactions including demethylation and hydroxylation. In addition, several lignans have been reported to modulate the activity of drug-metabolizing enzymes, highlighting their potential to participate in pharmacokinetic interactions with co-administered medications (Torres-Moreno et al., 2024). Excretion of lignans and their metabolites occurs primarily through the biliary and fecal routes, with a smaller proportion eliminated via the urinary tract. Understanding these metabolic pathways is essential for the rational design of pharmaceutical formulations to optimize systemic exposure and maximize the therapeutic potential of lignans.

Due to their lipophilic nature and structural diversity, the formulation of lignans remains a significant challenge in pharmaceutical development (Devi, 2019). These characteristics influence formulation design by affecting dissolution, chemical stability, membrane permeability, and drug release, thereby necessitating the development of specialized delivery systems to improve pharmaceutical performance.

The development of lignan-based formulations has progressed from traditional herbal preparations to more advanced drug delivery systems designed to address bioavailability limitations. For certain phytomedicines derived from Phyllanthus species (phyllanthin and hypophyllanthin) or Magnolia species (eudesmin and galgravin), standardized dry extracts have been incorporated into pharmaceutical dosage forms, including capsules, tablets, and coated tablets (Fang et al., 2018). These products are commonly manufactured using atomization or spray-drying techniques, in which liquid extracts are converted into fine powders, increasing the surface area available for dissolution and potentially improving dissolution in the gastrointestinal tract (Vidović et al., 2014). In addition to improving powder handling and manufacturing efficiency, these technologies contribute to the standardization of phytopharmaceutical preparations, facilitating dose uniformity, product stability, and quality control during industrial production.

Beyond conventional herbal preparations, advances in extraction technologies have enabled the production of highly purified lignan preparations with improved batch-to-batch consistency and pharmaceutical quality. These advances have also facilitated the development of innovative delivery platforms, including phytosomes, lipid-based carriers, and nanotechnology-based systems, designed to improve the stability, bioavailability, and therapeutic performance of lignans. Such technological approaches have the potential to improve lignan delivery and pharmaceutical performance; however, their safety and therapeutic efficacy should be evaluated according to the available preclinical and clinical evidence for each specific formulation. Numerous patents have been reported in these areas, reflecting ongoing efforts to improve the quality, stability, reproducibility, and biopharmaceutical properties of lignan-containing products.

Among the most important advances in phytopharmaceutical formulation is the development of phytosome technology. Unlike conventional liposomes, in which bioactive compounds are encapsulated within lipid vesicles, phytosomes are formed through the molecular complexation of bioactive constituents with phospholipids. In these systems, flavonolignans such as silymarin can form phyto-phospholipid complexes through interactions between their polar functional groups and the phospholipid head groups, potentially enhancing membrane affinity, facilitating intestinal permeation, and improving oral absorption and bioavailability (Semalty et al., 2010; De Iasi et al., 2017). Several studies have suggested that phytosomal formulations can improve the pharmacokinetic performance of poorly water-soluble phytochemicals compared with conventional plant extracts; however, the magnitude of these effects depends on the specific compound and formulation under investigation (Wang et al., 2018a). In addition, phytosomal formulations may reduce variability in oral absorption and improve the reproducibility of systemic exposure, although these effects should be confirmed for each lignan through well-designed pharmacokinetic and clinical studies.

Podophyllotoxin and its semisynthetic derivatives are among the most successful examples of pharmaceutical development from naturally occurring lignans (Ardalani et al., 2017). Although podophyllotoxin exhibits potent antineoplastic activity, its clinical application has been limited by poor aqueous solubility and considerable systemic toxicity. To overcome these limitations, structural modifications of the parent molecules led to the development of semisynthetic derivatives such as etoposide and teniposide. These compounds possess improved pharmaceutical properties, including enhanced aqueous solubility and a more favorable therapeutic index, facilitating their formulation as injectable dosage forms and their successful incorporation into clinical oncology practice. This example illustrates how structural modification, combined with appropriate pharmaceutical development, can transform naturally occurring lignans into clinically useful therapeutic agents while preserving their pharmacological activity (Bruneton, 2001).

Current research has increasingly focused on nanomedicine-based approaches to address the biopharmaceutical limitations associated with lignans. Lipid nanoparticles, nanoemulsions, and other nanocarrier systems have been investigated as platforms for encapsulating lignans such as conidendrin and tracheloside to improve their physicochemical stability, protect them from premature degradation, and enhance their oral bioavailability. In addition, these delivery systems may provide controlled drug release, improve encapsulation efficiency, enhance tissue distribution, and optimize pharmacokinetic performance. However, most of these formulations remain at the preclinical stage, and further studies are required to establish their safety, efficacy, and clinical applicability. Future studies should also address large-scale manufacturing, long-term stability, and regulatory considerations to facilitate their clinical translation.

**Table 2** summarizes the principal lignans of pharmaceutical interest, highlighting their pharmaceutical formulations, routes of administration, therapeutic applications, and current stage of development. Because these compounds are at different stages of pharmaceutical development, the level of available evidence varies considerably, ranging from approved drugs and dietary supplements to experimental formulations evaluated exclusively in preclinical studies. Accordingly, inclusion in **Table 2** should not be interpreted as evidence of equivalent clinical validation or regulatory approval, but rather as an overview of the current landscape of pharmaceutical development for lignan-based products.

**Table 2:** Pharmaceutical forms of lignans and their main therapeutic use.

Lignan	Pharmaceutical form	Route of administration	Therapeutic use	Development status	Reference
Podophyllotoxin	Gel	Topical	Genital warts caused by HPV	Clinical trial	(Bruneton, 2001)
Etoposide	Injectable Solution	IV	Cancer	Approved drug	(Goodman and Gilman, 2001)
Teniposide	Injectable Solution	IV	Cancer	Approved drug	(Brayfield, 2020)
Silybin A	Capsule	Oral	Hepatoprotective agent Prostate cancer	Phytomedicine	(European Union herbal monograph on Silybum marianum (L.) Gaertn., fructus. Committee on Herbal Medicinal Products (HMPC), 2018)
Silybin B	Capsule	Oral	Hepatoprotective	Phytomedicine	(European Union herbal monograph on Silybum marianum (L.) Gaertn., fructus. Committee on Herbal Medicinal Products (HMPC), 2018)
Isosylbin A	Tablet	Oral	Cirrhosis Hepatoprotective	Phytomedicine	(Féher and Lengyel, 2012)
Silychristin	Pills	Oral	Hepatoprotective	Phytomedicine	(Saller et al., 2001)
Silydianin	Oral Suspension	Oral	Hepatoprotective	Phytomedicine	(Saller et al., 2001)
Secoisolariciresinol	Powder	Oral	Dietary supplement and antioxidant	Phytomedicine	(Adlercreutz, 2007)
Enterodiol	Capsule	Oral	Antioxidant Anti-inflammatory	Phytomedicine	(Thompson et al., 1991)
Enterolactone	Capsule	Oral	Antioxidant Anti-inflammatory	Phytomedicine	(Thompson et al., 1991)
Arctiin	Capsule	Oral	Anti-inflammatory	Phytomedicine	(Bhatti et al., 2014)
Arctigenin	Mother Tincture	Oral	Antitumor Anti-inflammatory Antioxidant	Phytomedicine	(Bhatti et al., 2014)
Schisandrin A	Capsule	Oral	Dietary supplement	Phytomedicine	(Panossian and Wikman, 2008)
Schisandrol B	Capsule	Oral	Dietary supplement	Phytomedicine	(Panossian and Wikman, 2008)
Phyllanthin	Capsule	Oral	Antioxidant Hepatoprotective	Phytomedicine	(Mao et al., 2016)
Hypophyllanthin	Capsule	Oral	Hepatoprotective	Phytomedicine	(Wan Saidin et al., 2022)
Sesamin	Oil and Softgel Capsule	Oral	Anti-inflammatory Antioxidant	Dietary supplement	(Wu et al., 2019)
Sesamol	Cream	Topical	Antioxidant	Dietary supplement	(Wu et al., 2019)
Sesamolin	Soft Capsule	Oral	Hepatoprotective	Phytomedicine	(Wu et al., 2019)
Pinoresinol	Oil	Oral	Anti-inflammatoryAntioxidant	Dietary supplement	(Owen et al., 2000)
Lariciresinol	Capsule	Oral	Antitumor Anti-inflammatory Antioxidant	Phytomedicine	(Slaugh, 2002)
Magnolol	Gum and Toothpaste	Oral Care	Antimicrobial	Cosmetic application	(Lee et al., 2011)
Honokiol	Tablet and Injectable Solution	Oral/IV	Antimicrobial	Phytomedicine	(Lee et al., 2011)
Nordihydroguaiaretic acid (NDGA)	Ointment	Topical	Antioxidant, anti-inflammatory, antimicrobial, proposed application in skin infections	Experimental formulation (preclinical)	(Lambert et al., 2004)
Savinin	Capsule	Oral	Anti-inflammatory	Phytomedicine	(Cho et al., 2001)
Syringaresinol	Capsule	Oral	Anti-inflammatory	Phytomedicine	(Jang et al., 2022)
Isolariciresinol	Powder	Oral	Antioxidant	Phytomedicine	(Luzzani and Carreri, 2019)
Hydroxymatairesinol	Capsule	Oral	Antioxidant, anti-inflammatory	Phytomedicine	(Luzzani and Carreri, 2019)
Niranthin	Capsule	Oral	Antidiabetic Hepatoprotective	Phytomedicine	(Hidanah et al., 2022)
Tracheloside	Capsule	Oral	Anti-inflammatory	Phytomedicine	(고바야시 et al., 2012)
Matairesinol	Topical cosmetic formulations (creams, lotions, emulsions)	Topical	Skin moisturization, seboregulation, dermatological care	Cosmetic ingredient (patented applications)	(Renault and Catroux, 2004)
Episesamin	Soft Capsule	Oral	Antioxidant, anti-inflammatory	Phytomedicine	(Wu et al., 2019)
Medioresinol	Tablets	Oral	Antihypertensive	Phytomedicine	(Kim, 2015)
Asarinin	Ointment	Topical	Anti-inflammatory and anti-rheumatic	Phytomedicine	(Kang et al., 2020)
Shikokinin	Ointment	Topical	For Burns and Scalds	Phytomedicine	(Chen and Li, 2012)
Wuweizisu C	Nanoemulsion	Oral	Hepatoprotective	Experimental formulation (preclinical)	(Zhao et al., 2019)
Eudesmin	Tincture	Oral	Antioxidant, anti-inflammatory	Proposed therapeutic application	(He et al., 2019)
Galgratina	Capsule	Oral	Antioxidant, anti-inflammatory	Phytomedicine	(Sarrica et al., 2018)

Some of the biological activities and effects of the lignans reported in **Table 2** are discussed in greater detail in Section 5.

Despite significant progress in the development of pharmaceutical formulations, the large-scale application of lignans remains constrained by the limited availability of many natural sources and the relatively low yields from conventional extraction methods. Although formulation technologies have improved the physicochemical and biopharmaceutical performance of several lignans, ensuring a sustainable and reliable supply of these compounds remains a major challenge. Consequently, increasing attention has been paid to alternative production strategies that support pharmaceutical development while reducing pressure on natural plant populations.

The growing pharmacological, nutraceutical, and technological interest in lignans has increased the demand for production systems that integrate environmental sustainability, economic feasibility, and industrial scalability. Historically, direct extraction from plant biomass has been the primary method for obtaining these compounds (Patyra et al., 2022) and (Slanina and Glatz, 2004). However, the relatively low abundance of lignans in plant tissues, together with the slow growth and ecological vulnerability of key producer species, particularly those in the genus *Podophyllum*, limits the sustainable supply of these compounds. In addition, the environmental consequences associated with the overexploitation of plant genetic resources have further driven the search for alternative production strategies (Moraes et al., 2002); (Gulzar et al., 2024). As a result, increasing attention has been paid to biotechnological and sustainable approaches that support the large-scale production of lignans while reducing pressure on natural populations.

## 7 Sources of lignans: perspectives, limitations and challenges

### 7.1 Direct route: lignan extraction from plant biomass

Direct lignan extraction involves isolating secondary metabolites that were previously biosynthesized by plants, leveraging their specialized metabolism. In this strategy, lignans are recovered from complex plant matrices, where they accumulate differentially in specific organs or tissues, such as seeds, roots, rhizomes, or bark, and perform functions related to defense, signaling, and physiological regulation (Cacace and Mazza, 2006; Willför et al., 2006).

The extraction process typically begins with physical pretreatments (drying and particle-size reduction) that optimize mass transfer, followed by solid-liquid extraction with organic or hydroalcoholic solvents, selected based on the polarity and chemical state of the lignan (Slanina and Glatz, 2004; Patyra et al., 2022). In many cases, additional alkaline or enzymatic hydrolysis steps are required to release lignans either from their interactions with lignocellulosic cell-wall components or from naturally occurring macromolecular assemblies. In flaxseed, for example, secoisolaraciresinol diglucoside (SDG) occurs predominantly as part of an oligomeric lignan macromolecule, in which SDG units are ester-linked through 3-hydroxy-3-methylglutaric acid (HMGA) and associated with hydroxycinnamic acid glucosides. Therefore, hydrolysis is often required to depolymerize these structures and enable efficient lignan recovery (Kamal-Eldin et al., 2001; Struijs et al., 2008; Kezimana et al., 2018).

Historically, the isolation of these phenylpropanoid compounds was carried out using equilibrium techniques, such as maceration and percolation, with conventional organic solvents. Although these methods allow for appreciable yields, their operational kinetics are limited by resistance to intracellular mass transfer, which requires prolonged residence times and high consumption of volatile solvents. This compromises the selectivity of the process by co-extracting lipophilic interferents or pigments and generating critical volumes of xenobiotic waste that are costly to contain (Patyra et al., 2022). To overcome these limitations, bioprocess engineering has incorporated intensification technologies based on volumetric, rather than thermal, energy transfer, in line with the principles of green chemistry.

Among these technologies, ultrasound-assisted extraction (UAE) maximizes mass transfer coefficients through acoustic cavitation. The microlocal collapse of bubbles generates shock waves and high-shear microjets that induce mechanical rupture of cell walls, thereby facilitating the desorption and solubilization of the analyte via forced advection. This method has proven effective for the recovery of furanofuranolignans, such as sesamin and sesamolin, from *Sesamum indicum* seeds, achieving faster kinetics than conventional maceration (Devi, 2019). It has also been used for the selective extraction of dibenzocyclooctadienic lignans, such as schizandrine and deoxyschizandrine, from the fruit of *Schisandra chinensis*, preserving their thermolabile structures (Slanina and Glatz, 2004).

Microwave-assisted extraction (MAE) dissipates electromagnetic energy volumetrically through dipole rotation and ionic conduction. This localized heating causes vaporization of vacuolar water, generating a hydrostatic pressure gradient that promotes cell lysis and exponentially reduces the operating time and the solvent-to-biomass ratio. Notable examples include the isolation of secoisolariciresinol diglucoside (SDG) from flaxseed meal (*Linum usitatissimum*), where MAE facilitates its rapid desorption (Stücker et al., 2016), and the accelerated extraction of arctiin and arctigenin from *Arctium lappa* seeds (He et al., 2018). Supercritical fluid extraction (SFE-CO_2_) is a highly selective alternative for food and pharmaceutical applications. Operating above the critical point of carbon dioxide (Tc = 31.1 °C; Pc = 7.38 MPa), the fluid exhibits hybrid transport properties (quasi-gaseous diffusivity and quasi-liquid density). Its thermodynamic tuning, complemented by polar modifiers such as ethanol, enables selective fractionation of the lignan core without leaving traces of organic solvents in the final product. Using this platform, biphenyls such as honokiol and magnolol have been successfully obtained from the bark of *Magnolia officinalis*, guaranteeing residue-free extracts (Wan et al., 2015). Similarly, the ethanol-modified SFE has enabled the safe isolation of podophyllotoxin from Podophyllum biomass and *in vitro* tissue cultures, preventing its thermal degradation (Ardalani et al., 2017).

The integration of these technologies has led to sequential hybrid processes designed to synergistically maximize the yield and purity of phytopharmaceuticals. The subsequent processing of crude extracts is combined with advanced membrane separation operations (ultra- and nanofiltration) and preparative reversed-phase chromatography, ensuring the purity standards required by regulatory agencies (Yang et al., 2025).

An important advantage of direct extraction is that lignans are obtained with their naturally occurring stereochemistry already established, resulting from highly stereoselective biosynthetic pathways mediated by specialized enzyme complexes (Patyra et al., 2022). This characteristic eliminates the need for subsequent chiral resolution, which is typically costly and difficult to scale up in chemical synthesis (Schmidt et al., 2012; Tanase et al., 2019).

However, this strategy has structural limitations stemming from the low concentration of lignans in most plant species, which necessitates the processing of large volumes of biomass and reduces material and energy efficiency (Saarinen et al., 2007; Smeds et al., 2007; Patyra et al., 2022). Factors such as genetic variability, soil and climatic conditions, seasonality, and long growth cycles introduce fluctuations in yields and hinder industrial standardization (Chhillar et al., 2021; Rizzolo-Brime et al., 2022; Singh et al., 2024). From a sustainability perspective, this strategy involves high solvent consumption and intensive purification processes, with significant economic and environmental impacts. Therefore, its application is considered viable primarily in species with high lignan concentrations or in the absence of competitive synthetic, semi-synthetic, or biotechnological alternatives.

**Table 3** summarizes the main lignans identified in the literature, detailing the plant species and the specific tissues in which their greatest abundance has been reported. The data show that most of these compounds are concentrated in specific plant organs, primarily seeds and grains (e.g., *Linum usitatissimum*, *Sesamum indicum*, and *Arctium lappa*), followed by fruits, roots, and rhizomes (e.g., *Podophyllum* and *Schisandra chinensis*). This distribution provides a direct criterion for selecting the appropriate biomass based on the desired lignan.

**Table 3:** Lignans extracted directly from plants: abundance and main sources.

Lignan	Plant species with the highest reported abundance	Plant tissue and yield (mg/100 g)	Reference
Secoisolariciresinol diglucoside	*Linum usitatissimum*	Defatted seed (301-702)	(Tan et al., 2015; Zare et al., 2023)
Sesamin	*Sesamum indicum*	Seed (39-370)	(Ghotbzadeh Kermani et al., 2019)
Sesamolin	*Sesamum indicum*	Seed (15-20)	(Myint et al., 2020)
Podophyllotoxin	*Podophyllum hexandrum*, *P. peltatum*	Rhizome, root (100-500)	(Sharma et al., 2000; Hemant et al., 2015)
Schisandrin/Gomisin A	*Schisandra chinensis*	Fruit (50-250)	(Szopa et al., 2018)
Arctigenin	*Arctium lappa*	Seed (200-600)	(Gao et al., 2018)
Syringaresinol	*Eleutherococcus senticosus*	Bark, grain, root (10-40)	(Patyra et al., 2025)
Pinoresinol	*Whole grains, oilseeds*	Grain/seed (5-70)	(Milder et al., 2005)
Lariciresinol	*Cereals, legumes*	Grain (10-70)	(Durazzo et al., 2013)
Matairesinol	*Vegetables and various fruits*	Miscellaneous (20-50)	(Niemeyer et al., 2003)

### 7.2 Synthetic routes

From the perspective of organic synthesis, lignans are particularly complex molecular targets due to their high functional density, multiple stereogenic centers, and wide structural diversity, ranging from relatively simple skeletons (dibenzylbutanes and furofurans) to highly complex systems such as aryltetralin and dibenzocyclooctadiene lignans. The efficient construction of these structures requires precise control of the chemo-, regio-, and stereochemistry throughout the entire synthetic route, thereby making lignans paradigmatic models for the development of advanced methodologies in natural product chemistry (Raghavendra et al., 2016; Fang and Hu, 2018).

The determining step in most synthetic strategies is the formation of the C8–C8′ linkage, which defines the characteristic C6–C3–C3–C6 architecture through the coupling of two phenylpropanoid units (Soorukram et al., 2018). Traditionally, this bond has been constructed using convergent carbon-carbon bonding strategies, such as aldol condensations or malonate-based transformations, which generate dibenzylic skeletons susceptible to cyclization or further oxidation (Wang et al., 2006). Although these approaches are robust and suitable for lignans with relatively simple structures, they often offer limited stereoselective control, leading to racemic mixtures that require additional resolution steps, reducing overall yield and frequently resulting in cumulative process yields below 10% for highly functionalized targets (Enders et al., 2002).

To approximate the efficiency of plant biosynthesis, contemporary strategies have incorporated oxidative couplings, allowing the direct formation of the C8–C8′ bond and reducing the number of transformations required (Zi et al., 2018; Li et al., 2019). However, in the absence of directional control equivalent to that exerted by plant proteins, these reactions typically exhibit low regio- and stereoselectivity, limiting their application in the synthesis of lignans with high stereostructural definition (Soorukram et al., 2018).

Once the core backbone is established, the complexity of the synthetic pathway depends on the lignan’s structural type (Ward, 2003). Furofuran-type lignans are typically obtained through thermodynamically favorable intramolecular cyclizations, whereas aryltetraline lignans, such as podophyllotoxin, require highly controlled sequential ring closures, often via electrophilic Friedel-Crafts substitutions or oxidative rearrangements (Syed et al., 2014; Ting and Maimone, 2014; Raghavendra et al., 2017). While these transformations have high academic and methodological value, they considerably reduce practical feasibility and the potential for industrial scale-up.

Since the biological activity of many lignans depends critically on their absolute configuration, asymmetric synthesis plays a central role in this field. The use of chiral auxiliaries, organocatalytic strategies, and chirality-transfer processes has enabled access to pure enantiomers, which are essential for structure-activity relationship studies and preclinical development ( (Zi et al., 2018; Li et al., 2019); (Zi et al., 2018; Li et al., 2019); (Beaupérin et al., 2017; Hao and Xu, 2024). However, the technical complexity, operational sensitivity, and costs associated with these methodologies remain significant limitations to their large-scale implementation (Ward, 2003).

From a practical and industrial perspective, however, the applicability of total lignan synthesis remains limited despite remarkable methodological advances. The synthesis of complex aryltetralin lignans, particularly podophyllotoxin and related derivatives, often requires lengthy multistep sequences, rigorous stereochemical control, and multiple purification procedures, resulting in poor step economy and reduced overall process efficiency (Shen et al., 2022; Flores-Hernández et al., 2024). Although modern asymmetric methodologies have significantly improved access to these targets, cumulative yields frequently remain modest, especially for highly functionalized lignans, while the operational complexity and resource-intensive nature of these routes hinder their scalability and large-scale implementation (Enders et al., 2002; Shen et al., 2022). Recent reviews emphasize that total synthesis is particularly valuable for structural confirmation, mechanistic studies, and the preparation of novel analogues; however, commercial production is generally more effectively supported by semisynthetic or emerging biosynthetic approaches (Shen et al., 2022; Xianggu et al., 2024). Consequently, while total synthesis continues to play a pivotal role in lignan research and methodological innovation, its economic attractiveness for large-scale manufacturing remains limited when naturally occurring precursors are available, making semisynthesis the preferred strategy for the production of many bioactive lignan derivatives (Flores-Hernández et al., 2024; Xianggu et al., 2024).

### 7.3 Semisynthesis routes

Semisynthesis is widely regarded as a practical strategy for lignan development because it leverages the structural complexity and stereochemical definition produced by plant biosynthesis, while enabling selective chemical diversification (López-Rojas et al., 2022; Plaha et al., 2022). In contrast to total synthesis, which necessitates the *de novo* assembly of complex molecular architectures, semisynthetic methods begin with naturally occurring lignans. As a result, these approaches typically involve fewer synthetic steps, enhance process efficiency, and offer greater potential for industrial application (Botta et al., 2001; Mueed et al., 2023). This strategy has proven especially effective for optimizing pharmacological and physicochemical properties while maintaining the integrity of the bioactive scaffold (Alvarenga et al., 2021; López-Rojas et al., 2022).

Selective oxidations, glycosylations, and aromatic substitutions of podophyllotoxin have yielded derivatives such as etoposide and teniposide, which exhibit enhanced therapeutic indices and a distinct mechanism of action that involves inhibition of topoisomerase II (Kim et al., 2020; Arora et al., 2024). This demonstrates that semisynthesis can yield clinically valuable agents without the need to reconstruct complex stereochemistry.

In addition to podophyllotoxin, semisynthetic modifications of arctigenin, matairesinol, hinokinin, and glycosylated lignans have enhanced anti-inflammatory, antiviral, antiproliferative, and bioavailability-related properties (Botta et al., 2001; Boldizsár et al., 2010; Consonni and Ottolina, 2022; López-Rojas et al., 2022). These results underscore the versatility of peripheral functional-group modification for optimizing lignans.

Compared to total synthesis, semisynthesis preserves biologically defined stereochemistry, typically requires fewer transformations, and is more easily adapted to industrial production (Umezawa et al., 2011; Satake et al., 2015; Soorukram et al., 2018). However, its applicability may be limited by dependence on natural sources, supply variability, and the occasional need for protection-deprotection strategies (Yousefzadi et al., 2010; Satake et al., 2013).

In summary, semisynthesis is one of the most practical approaches for lignan optimization, as it integrates the stereochemical complexity of natural products with the adaptability of chemical modification. Examples such as etoposide, teniposide, arctigenin, matairesinol, and hinokinin derivatives illustrate that targeted transformations can substantially enhance biological activity, selectivity, and pharmacokinetic properties. Although limitations in precursor availability persist, semisynthesis is often the preferred industrial strategy due to its efficiency, scalability, and cost-effectiveness (Umezawa et al., 2011; Satake et al., 2015; López-Rojas et al., 2022).

### 7.4 Production by plant biotechnology

#### 7.4.1 *In vitro* plant tissue cultures as platforms for lignan production

Although certain cultivated species, such as flax (*L. usitatissimum*) and sesame (*S. indicum*), are recognized as major commercial sources of lignans due to their relatively high accumulation of secoisolariceresinol diglucoside and sesamin, respectively (Schmidt et al., 2012; Ghotbzadeh Kermani et al., 2019; Chhillar et al., 2021; Arab et al., 2022), many other lignan-producing plants exhibit low metabolite yields, restricted geographic distribution, slow growth rates, or limited biomass availability. Furthermore, several lignans of high pharmaceutical interest, particularly podophyllotoxin and related aryltetralin derivatives, are produced in only a few species and often occur at low concentrations. Consequently, despite the availability of abundant lignans, production remains constrained by biological and agronomic factors. Together with the considerable structural complexity of these compounds, these limitations have stimulated the development of plant biotechnology approaches, particularly *in vitro* culture systems, as attractive alternatives to conventional plant extraction and total chemical synthesis.

Among the genera studied for lignan production, Linum is one of the most extensively investigated because of its importance as a model system for lignan biosynthesis and plant biotechnology. This importance stems from its diverse lignan profile and the availability of well-established biotechnological protocols (Ionkova et al., 2010; Renouard et al., 2018).

From a functional perspective, each culture platform differs in its degree of cellular organization and, therefore, in its productivity, stability, and experimental flexibility. Callus cultures constitute the most undifferentiated state; cell suspensions are typically derived from friable calli and offer greater physiological homogeneity; and hairy roots retain more root differentiation traits that can be crucial for the sustained expression of complex biosynthetic pathways (Ionkova et al., 2010; Renouard et al., 2018; Elbouzidi et al., 2026).

A systematic, quantitative analysis of the available literature on the biosynthesis of these secondary metabolites is presented below. The plant families most extensively studied biotechnologically for enhancing lignan biosynthesis are Linaceae, Euphorbiaceae, Oleaceae, Apiaceae, Berberidaceae, and Cupressaceae, and include specifically species such as *Linum usitatissimum* (Nadeem et al., 2018, 2019; Abbasi et al., 2019), *Linum album* (Smollny et al., 1998; Baldi et al., 2008; Tahsili et al., 2014), *Linum nodiflorum* (Konuklugil et al., 1999), *Podophyllum hexandrum* (Chattopadhyay et al., 2002; Anrini and Jha, 2009; Bhattacharyya et al., 2012), *Anthriscus sylvestris* (Koulman et al., 2003), *Schisandra henryi* (Jafernik et al., 2024a), *Forsythia koreana* (Murata et al., 2015), *Juniperus chinensis* (Premjet and Tachibana, 2004), *Juniperus virginiana* (Kašparová et al., 2018) and *Phyllanthus niruri* (Elfahmi et al., 2006). **Table 4** presents, in a structured manner, the yields and accumulation profiles of specific lignans, categorized by plant species and the *in vitro* culture platform used; the chemical structures of lignans found by the *in vitro* culture system are previously shown in **Table 1**. These data allow direct comparison of lignan production among callus cultures, cell suspension cultures, and hairy root systems.

**Table 4:** Lignans reported in callus cultures, suspension cells and hairy roots.

Lignan	Plant species	Vegetable tissue culture					Reference
		Callus	Suspension cell	Hairy root	Transformed hairy root	Yield (mg/g DW)	
Secoisolariciresinol diglucoside	*Linum usitatissimum*	✓	✓			2.23-5.55	(Bose et al., 2020)
		✓				5.5-34.26	(Jalal et al., 2022)
		✓	✓			18.42	(Seta-Koselska and Skórzyńska-Polit, 2017; Ahmad et al., 2019; Markulin et al., 2019, 2021)
				✓	✓	7.1	(Anjum et al., 2020)
					✓		(Gabr et al., 2016, 2018)
	*Linum grandiflorum*	✓			✓	4.5-8.0	(Asad et al., 2021)
Lariciresinol diglucoside	*Linum grandiflorum*	✓			✓	2.0-5.0	(Asad et al., 2021)
	*Linum usitatissimum*	✓	✓		✓	1.0-4.5	(Bose et al., 2020)
		✓	✓	✓	✓	11.9	(Anjum et al., 2020)
		✓			✓		(Gabr et al., 2016, 2018)
		✓	✓		✓		(Seta-Koselska and Skórzyńska-Polit, 2017; Ahmad et al., 2019; Markulin et al., 2019, 2021)
Matairesinol	*Forsythia x intermedia*	✓	✓		✓	2.24	(Schmitt and Petersen, 2002)
	*Linum flavum*	✓	✓	✓	✓		(Wichers et al., 1991; Mikac et al., 2021)
	*Linum persicum*	✓	✓	✓	✓		(Esfandiari et al., 2018)
	*Linum usitatissimum*	✓	✓	✓	✓		(Gabr et al., 2016, 2018)
		✓	✓	✓	✓		(Bose et al., 2020)
Dehydrodiconiferyl alcohol glucoside and Guaiacylglycerol-β-coniferyl alcohol ether glucoside	*Linum grandiflorum*	✓			✓		(Asad et al., 2021)
	*Linum usitatissimum*	✓		✓	✓	DCG: 21.6, GGCG: 4.9	(Anjum et al., 2020)
		✓		✓	✓		(Gabr et al., 2016, 2018)
		✓	✓	✓	✓	DCG: 58.85,	(Seta-Koselska and Skórzyńska-Polit, 2017; Ahmad et al., 2019; Markulin et al., 2019, 2021)
Podophyllotoxin	*Linum flavum*	✓			✓	0.6	(Mikac et al., 2021)
	*Linum album*	✓	✓		✓		(Smollny et al., 1998; Yousefzadi et al., 2010)
	*Juniperus chinensis*	✓	✓		✓		(Premjet and Tachibana, 2004)
	*Linum linearifolium*	✓	✓		✓	5.38	(Ionkova et al., 2010)
	*Dysosma pleiantha*	✓			✓		(Karuppaiya and Tsay, 2020)
	*Linum mucronatum ssp. mucronatum*	✓			✓	4.55	(Samadi et al., 2014)
	*Podophyllum hexandrum*	✓	✓		✓		(Chattopadhyay et al., 2002; Lin et al., 2003)
	*Podophyllum peltatum*	✓	✓		✓		(Anbazhagan et al., 2008)
	*Anthriscus sylvestris*	✓	✓		✓		(Koulman et al., 2003)
	*Juniperus procera*	✓	✓		✓		(Salih et al., 2021)
6-methoxy podophyllotoxin	*Linum mucronatum ssp. mucronatum*	✓			✓	41.38	(Samadi et al., 2014)
	*Linum album*	✓			✓	37.0-48.0	(Chashmi et al., 2013; Lalaleo et al., 2016)
	*Linum persicum*	✓	✓		✓	40.0-56.0	(Esfandiari et al., 2018)
	*Linum flavum*	✓	✓		✓	8.4	(Mikac et al., 2021)
	*Linum mucronatum*	✓	✓		✓	41.38	(Konuklugil et al., 2001)
Pinoresinol	*Linum flavum*	✓			✓		(Mikac et al., 2021)
	*Forsythia x intermedia*	✓			✓	0.86	(Schmitt and Petersen, 2002)
	*Ipomea cairica*	✓	✓		✓		(Páska et al., 2002)
Sesamin	*Sesamum indicum*	✓	✓		✓	0.063-0.46	(Wadhwa et al., 2023)
	*Forsythia*	✓	✓		✓		(Murata et al., 2015)
	*Zanthoxylum zanthoxyloides*	✓	✓		✓		(Etsè et al., 2014)
Cubein methylether	*Forsythia*	✓ g	✓				(Kim et al., 2009)
deoxypodophyllotoxin	*Podophyllum hexandrum*	✓	✓				(Chattopadhyay et al., 2002)
5’-demethoxy-6-methoxypodophyllotoxin	*Linum nodiflorum*	✓					(Berim et al., 2005)
Urinatetralin	*Phyllanthus niruri*	✓	✓				(Elfahmi et al., 2006)

A check mark (✓) indicates the specific *in vitro* culture system (callus culture, cell suspension culture, hairy root culture, or transformed hairy root culture) in which the corresponding lignan was reported in the cited reference.

#### 7.4.2 Callus cultures: establishment, plasticity, and productive limitations

Callus cultures serve as the primary platform for establishing and selecting productive cell lines in most lignan-producing species. The formation of callus involves the dedifferentiation of somatic cells into proliferating, unorganized tissue and is influenced by the morphogenic competence of the explant, effective sterilization procedures, and the balance of phytohormones in the culture medium (Ikeuchi et al., 2013; Xu et al., 2018; Nikule et al., 2020; Maqbool et al., 2025). In *Linum* species, callus induction is typically achieved using combinations of auxins (2,4-D, NAA, or IAA) and cytokinins such as kinetin (Ionkova et al., 2010).

Multiple studies have confirmed that callus cultures can accumulate lignans. In *Linum usitatissimum*, reported levels of secoisolariciresinol diglucoside (SDG) range from 2.23–5.55 mg/g to 5.5–34.26 mg/g dry weight (DW), while *L. grandiflorum* accumulates 4.5–8.0 mg/g DW of SDG (Jalal et al., 2022; Bose et al., 2020; Asad et al., 2021). Lariciresinol diglucoside (LDG) has been detected at concentrations of 2.0–5.0 mg/g DW in *L. grandiflorum* and 1.0–4.5 mg/g DW in *L. usitatissimum*. Additionally, callus cultures of *L. linearifolium* have produced 5.38 mg/g DW of podophyllotoxin (Ionkova et al., 2010; Bose et al., 2020; Asad et al., 2021).

The primary advantage of callus cultures is their flexibility, which enables the selection of productive genotypes, the optimization of culture conditions, assessment of hormonal effects, and the production of starting material for cell suspensions and transformed root cultures (Ionkova et al., 2010). However, this flexibility is frequently associated with lower metabolic stability and reduced lignan accumulation compared to more differentiated systems.

For example, in *L. linearifolium*, higher kinetin concentrations stimulated cell proliferation but decreased secondary metabolite accumulation, demonstrating a trade-off between biomass production and lignan biosynthesis (Ionkova et al., 2010). Consequently, while callus cultures can produce notable quantities of lignans, their primary value lies in the establishment, selection, and optimization of high-producing lines rather than in large-scale metabolite production (Ionkova et al., 2010; Gabr et al., 2016).

#### 7.4.3 Cell suspensions: physiological homogeneity and modulation of biosynthetic flux

Cell suspension cultures originate from friable callus tissues that are transferred to agitated liquid media. Compared with callus cultures, these systems offer enhanced control over growth conditions, nutrient availability, and elicitor treatments. This increased control makes cell suspension cultures a valuable platform for investigating lignan biosynthesis and optimizing metabolite production (Perez-Matas et al., 2023) (Berim et al., 2005).

Among various *in vitro* systems, cell suspensions have achieved some of the highest reported lignan yields. For example, in *Linum usitatissimum*, concentrations reached up to 18.42 mg/g dry weight (DW) for secoisolariciresinol diglucoside (SDG) and 28 mg/g DW for lariciresinol diglucoside (LDG). In other species, *Forsythia* × intermedia yielded 2.24 mg/g DW of matairesinol, while *Linum linearifolium* produced 5.38 mg/g DW of podophyllotoxin (Schmitt and Petersen, 2002; Ionkova et al., 2010; Seta-Koselska and Skórzyńska-Polit, 2017; Ahmad et al., 2019; Markulin et al., 2021). Additionally, cell suspensions have been shown to accumulate substantial amounts of glycosylated and methoxylated lignan derivatives (Ahmad et al., 2019; Bose et al., 2020).

A primary advantage of cell suspensions is their responsiveness to elicitation. Biological, hormonal, and abiotic elicitors can markedly enhance lignan production by activating defense-related pathways and redirecting metabolic flux. In *L. usitatissimum*, treatments with yeast extract and chitosan increased the accumulation of SDG and LDG, demonstrating the effectiveness of pathogen-derived signals in stimulating lignan biosynthesis (Nadeem et al., 2018; Ahmad et al., 2019).

Hormonal elicitors have demonstrated efficacy in modulating lignan biosynthesis. Salicylic acid promoted the accumulation of lignans and neolignans, while methyl jasmonate favored the production of pharmaceutically important aryltetralin lignans, such as 6-methoxypodophyllotoxin and related compounds. These findings underscore the capacity of plant signaling molecules to regulate specific branches of lignan biosynthesis (Anjum et al., 2017; Abbasi et al., 2019; Zahir et al., 2019).

Abiotic elicitors can further augment lignan accumulation. For instance, UV-C irradiation increased SDG production, while silver and zinc oxide nanoparticles induced stress responses that correlated with elevated lignan levels. In certain studies, nanoparticle treatments resulted in combined SDG and LDG yields exceeding 60 mg/g DW, among the highest reported for lignan-producing cell suspensions (Zahir et al., 2018; Abbasi et al., 2019) (Nadeem et al., 2018).

Mechanistic studies corroborate these observations. In *Linum nodiflorum*, elicitation increased the accumulation of aryltetralin lignans and stimulated the activity of enzymes involved in the final steps of podophyllotoxin biosynthesis, such as deoxypodophyllotoxin-6-hydroxylase and β-peltatin-6-O-methyltransferase. These results indicate that cell suspensions are valuable not only for metabolite production but also for investigating the regulation of lignan biosynthetic pathways (Berim et al., 2005).

In summary, cell suspensions offer a flexible platform for lignan production and metabolic engineering. Their rapid response to elicitors and the ability to precisely control culture conditions make them especially advantageous for optimizing biosynthetic pathways and enhancing lignan yields (Berim et al., 2005; Nadeem et al., 2018; Ahmad et al., 2019).

However, the performance of cell suspensions depends heavily on the cell line, physiological state, and culture conditions. Furthermore, the lack of tissue organization can restrict the long-term accumulation of metabolites typically associated with differentiated organs. Consequently, while cell suspensions are highly effective for mechanistic studies and metabolic optimization, their industrial application may be limited by challenges related to stability and process robustness (Berim et al., 2005; Khalafalla, 2026).

#### 7.4.4 Hairy roots: organ-specific stability and high productivity for aryltetraline lignans

Hairy roots, generated via Agrobacterium *rhizogenes*, represent the most differentiated system among the *in vitro* culture platforms discussed. These cultures exhibit rapid growth, genetic stability, and the capacity to proliferate in hormone-free media. Compared with undifferentiated cultures, hairy roots typically maintain more stable metabolic profiles and are particularly effective at producing compounds associated with root-specific biosynthetic pathways (Renouard et al., 2018; Morey and Peebles, 2022).

High lignan productivity has been observed in hairy root cultures of several Linum species. For example, concentrations of 6-methoxypodophyllotoxin reached 41.38 mg/g dry weight (DW) in *Linum mucronatum*, 37–48 mg/g DW in *L. album*, and 40–56 mg/g DW in *L. persicum*. In *L. flavum*, 8.4 mg/g DW of 6-methoxypodophyllotoxin was reported, while *L. mucronatum* ssp. *mucronatum* accumulated 4.55 mg/g DW of podophyllotoxin. Additionally, hairy roots of *L. usitatissimum* produced 7.1 mg/g DW of secoisolariciresinol diglucoside (SDG) and 11.9 mg/g DW of lariciresinol diglucoside (LDG) (Samadi et al., 2014; Gabr et al., 2016, 2018; Esfandiari et al., 2018; Anjum et al., 2020; Mikac et al., 2021).

A primary advantage of hairy roots is their capacity to maintain stable root-specific metabolic pathways while supporting high lignan production. For instance, transformed root lines of *L. flavum* accumulated deoxypodophyllotoxin, podophyllotoxin, and 6-methoxypodophyllotoxin. In this species, cultivation in Gamborg B5 medium with methyl jasmonate or ferulic acid supplementation further enhanced lignan accumulation (Renouard et al., 2018). Comparable outcomes have been documented in other species. In *Hyptis suaveolens*, optimization of the culture medium, thiamine supplementation, and improved extraction procedures increased podophyllotoxin production to 5.6 mg/g DW, surpassing levels found in non-transformed roots and wild plants (Bazaldúa et al., 2019).

Although hairy roots respond to elicitors, increases in lignan accumulation are often less pronounced than those observed in cell suspension cultures. In *Taxus × media* and *Linum album*, elicitation with precursors, methyl jasmonate, fungal extracts, or extracellular coniferaldehyde resulted in only modest increases in lariciresinol diglucoside and podophyllotoxin accumulation (Esmaeilzadeh Bahabadi et al., 2014; Ahmadian Chashmi et al., 2016; Sykłowska-Baranek et al., 2018). These findings indicate that elicitor effectiveness in hairy roots is strongly influenced by the species, the target metabolite, and the culture’s physiological state. In summary, hairy roots offer high genetic stability and sustained lignan production, positioning them as one of the most promising platforms for aryltetralin lignan production. Although their response to elicitation is less consistent than that of cell suspensions, their high basal productivity and long-term stability provide significant advantages for biotechnological applications (Renouard et al., 2018; Morey and Peebles, 2022). **Table 5** details and systematizes the impact of the main elicitor agents applied on the productive efficiency of lignans in these tissue and cellular platforms.

**Table 5:** Elicitors used in plant tissue cultures that produce lignans.

Lignan	Elicitor	Plant specie	Vegetable tissue culture	Yield (mg/g DW)	Reference
Secoisolariciresinol diglucoside	Yeast-extract	*Linum usitatissimum*	Cell suspension cultures	10.1	(Nadeem et al., 2018)
	Chitosan			18.42	(Ahmad et al., 2019)
	Silver nanoparticles			67.23	(Zahir et al., 2018)
	Zinc oxide nanoparticles			2.98	(Abbasi et al., 2019)
	UV-C radiations			7.1	(Anjum et al., 2017)
	Salicylic acid			7.95	(Nadeem et al., 2018)
	Fungal mycelium *Botrytis cinerea*, *Phoma exigua* and *Fusarium oxysporum* F *ssp lini*				(Hano et al., 2020)
	*Fusarium oxysporum*	*Linum usitatissimum*	Hairy roots		(Markulin et al., 2019)
	*L*-Phenylalanine, coniferyl alcohol, methyl jasmonate	*Taxus x media*	Hairy roots		(Sykłowska-Baranek et al., 2018)
Lariciresinol diglucoside	Yeast-extract	*Linum usitatissimum*	Cell suspension cultures	11.0	(Nadeem et al., 2018)
	Chitosan			28.0	(Ahmad et al., 2019)
	Silver nanoparicles			67.23	(Zahir et al., 2019)
	Zinc oxide nanoparticles			3.86	(Abbasi et al., 2019)
	UV-C radiations				(Anjum et al., 2017)
	Salicylic acid				(Nadeem et al., 2019)
	*L*-Phenylalanine, coniferyl alcohol, methyl jasmonate	*Taxus x media*	Hairy roots	0.00319	(Sykłowska-Baranek et al., 2018)
	Extracellular coniferaldehyde	*Linum album*	Hairy root	0.107	(Ahmadian Chashmi et al., 2016)
	Abscisic acid	*Linum usitatissimum*	Hairy root		(Renouard et al., 2012, 2018)
	*Fusarium graminearum* extract	*Linum album*	Hairy root	0.0136	(Esmaeilzadeh Bahabadi et al., 2014)
	Methyl jasmonate	*Isatis indigotica*	Hairy root	0.3539	(Chen et al., 2015)
Dehydrodiconiferyl alcohol glucoside	Silver nanoparticles	*Linum usitatissimum*	Cell suspension cultures		(Zahir et al., 2019)
	Zinc oxide nanoparticles			37.10	(Abbasi et al., 2019)
	UV-C radiations				(Anjum et al., 2017)
	Salicylic acid			54.9	(Nadeem et al., 2019)
Guaiacylglycerol-β-coniferyl alcohol ether glucoside	Silver nanoparticles	*Linum usitatissimum*	Cell suspension cultures	45.9	(Zahir et al., 2019)
	Zinc oxide nanoparticles			2.91	(Abbasi et al., 2019)
	UV-C radiations			9.2	(Anjum et al., 2017)
	Salicylic acid			16.78	(Nadeem et al., 2019)
Pinoresinol	*L*-Phenylalanine, coniferyl alcohol, methyl jasmonate	*Taxus x media*	Hairy roots		(Sykłowska-Baranek et al., 2018)
	Extracellular coniferaldehyde	*Linum album*	Hairy root		(Ahmadian Chashmi et al., 2016)
Podophyllotoxin	Extracellular coniferaldehyde	*Linum album*	Hairy root	0.0087	(Ahmadian Chashmi et al., 2016)
	*Fusarium graminearum* extract			0.0187	(Esmaeilzadeh Bahabadi et al., 2014)
	Culture filtrate of *Piriformospora indica*				(Kumar et al., 2012; Tashackori et al., 2016)
	Salicylic acid	*Juniperus virginiana*	Suspension cell cultures	0.15	(Kašparová et al., 2018)
6-methoxy podophyllotoxin	*Trichoderma viride* extract	*Linum album*	Hairy root		(Esmaeilzadeh Bahabadi et al., 2014)
	Culture filtrate of *Piriformospora indica*				(Kumar et al., 2012; Tashackori et al., 2016)
	Methyl jasmonate	*Linum album*	Cell suspension cultures	36.19	(van Fürden et al., 2005)

#### 7.4.5 Industrial scaling up lignan-producing plant tissue cultures: challenges, limitations, and possibilities

The transition from laboratory-scale to industrial-scale production of lignan-producing plant tissue cultures remains a significant challenge for commercial application. While plant cell, tissue, and organ cultures represent renewable sources of high-value lignans, few plant biotechnology processes have demonstrated both technical feasibility and economic viability at the industrial level (Titova et al., 2024).

Scale-up encompasses more than simply increasing culture volume. Critical parameters—including bioreactor design, operating mode (batch, fed-batch, continuous, or semi-continuous), aeration, mixing, and nutrient supply—must be optimized to sustain cell growth and metabolite production. Due to the wide variation in physiological and physical characteristics among plant cultures, scale-up strategies must be customized for each species and production system (Titova et al., 2024) (Motolinía-Alcántara et al., 2021).

A primary limitation encountered during scale-up is oxygen transfer. Plant cells generally exhibit slow growth, tend to form aggregates, and display high sensitivity to microenvironmental changes. As reactor volume increases, ensuring sufficient oxygen availability becomes increasingly challenging, which can adversely impact both biomass accumulation and lignan production (Verdú-Navarro et al., 2023; Titova et al., 2024). Additionally, agitation and aeration may induce mechanical stress, leading to cell damage and reduced productivity (Verdú-Navarro et al., 2023; Titova et al., 2024).

Other significant challenges involve aggregate formation, foam generation, the development of nutrient and oxygen gradients, and difficulties in maintaining uniform culture conditions within large bioreactors (Verdú-Navarro et al., 2023; Häkkinen et al., 2024; Titova et al., 2024). These factors are especially critical in lignan-producing cultures, as lignan biosynthesis is frequently affected by the cells’ physiological state and the activation of stress-responsive metabolic pathways (Motolinía-Alcántara et al., 2021); (Verdú-Navarro et al., 2023; Titova et al., 2024).

In callus and suspension cultures, productivity often declines over time due to biological instability. High-yielding cell lines may gradually lose their biosynthetic capacity during repeated subculturing or after alterations in culture conditions. Additional commonly reported limitations include slow growth rates, genetic instability, inadequate control of differentiation, and increased contamination risks during prolonged cultivation periods (Khalafalla, 2026).

Hairy root cultures typically exhibit greater genetic and metabolic stability compared to undifferentiated systems and sustain elevated levels of organ-specific metabolite production (Liu et al., 2025). However, scaling up these cultures introduces distinct challenges. Dense root networks can impede oxygen and nutrient diffusion, resulting in internal mass-transfer limitations that diminish growth and productivity. Therefore, specialized low-shear and root-adapted bioreactor designs are frequently necessary (Khan et al., 2019).

No universal bioreactor design is suitable for all lignan-producing cultures. Stirred-tank, airlift, wave, and single-use reactors have been successfully employed for plant cell cultures; however, their effectiveness depends on factors such as aggregate size, oxygen requirements, and sensitivity to mechanical stress (Verdú-Navarro et al., 2023; Titova et al., 2024); (Motolinía-Alcántara et al., 2021)). For hairy root cultures, additional systems such as bubble-column, trickle-bed, nutrient-mist, and hybrid reactors have been developed to enhance oxygen transfer and biomass distribution (Khan et al., 2019).

Economic feasibility is a critical consideration in large-scale plant tissue culture production. Extended cultivation periods, stringent aseptic conditions, continuous reactor operation, and the maintenance of stable, high-producing lines are required. These factors collectively elevate production costs and have contributed to the limited number of commercially successful cases reported to date ( (Verdú-Navarro et al., 2023; Titova et al., 2024); (Khalafalla, 2026)).

Despite these challenges, plant tissue cultures offer significant advantages for producing high-value lignans. These systems enable controlled production conditions, reduce reliance on field cultivation, and offer sustainable alternatives when natural resources are scarce or extraction poses environmental challenges (Wu et al., 2021). Furthermore, plant cultures preserve native biosynthetic pathways and support the production of structurally complex metabolites (Bapat et al., 2023). Advances in bioreactor design, process automation, online monitoring, metabolic engineering, and synthetic biology are improving the prospects for large-scale lignan production. These developments are expected to enhance culture stability, increase productivity, and improve process control, bringing plant tissue culture systems closer to industrial implementation ( (Verdú-Navarro et al., 2023; Titova et al., 2024); (Verdú-Navarro et al., 2023; Häkkinen et al., 2024; Titova et al., 2024)). ( (Liu et al., 2025)).

To facilitate the selection of an appropriate production platform, **Table 6** provides a comparative overview of the main biological, operational, and technological characteristics of callus culture, cell suspensions, adventitious roots, and hairy roots, highlighting their respective strengths and limitations for lignan biomanufacturing.

**Table 6:** Comparative analysis of biological and operational variables for the strategic selection of lignan biomanufacturing platforms.

Characteristic	Callus culture	Cell suspension	Adventitious roots	Hairy roots
Level of organization	Undifferentiated tissue	Isolated cells, small aggregates	Differentiated organ (root)	Transformed differentiated organ
Origin	Explants (leaf, stem, etc.)	Derived from friable callus	Auxin-induced	Transformation with *Agrobacterium rhizogenes*
Phytohormone requirement	Yes	Yes	Yes	No (autonomous growth)
Genetic stability	Low to medium	Medium	Medium-high	High
Metabolic stability	Low (high variability)	Medium (line-dependent)	Medium-high	High
Growth rate	Moderate	High	Moderate	High
Culture homogeneity	Low	High	Medium	Medium
Lignan accumulation capacity	Moderate	Moderate-high (with elicitation)	High (vs. callus)	Very high
Representative application in *Linum*	Podophyllotoxin production in callus (*L. linearifolium*)	Elicitor-induced production	Greater accumulation than Callus (*L. usitatissimum*)	High levels in *L. flavum* and L. *mucronatum*
Response to elicitors	Medium	Very high	Medium	High
Ease of establishment	High	Medium	Medium	Low (requires transformation)
Ease of scaling up	Limited	High (liquid bioreactors)	Medium	High (but complex technique)
Regulatory considerations	Favorable	Favorable	Favorable	More restrictive (GMOs)
Main application	Line selection	Metabolic optimization	Non-transgenic production	Stable high-yield production

In summary, successful industrial production of lignans requires balancing biological performance, engineering demands, and economic feasibility. While challenges such as culture stability, oxygen transfer, and production costs persist, ongoing advancements in bioprocess engineering and metabolic optimization reinforce the potential of plant tissue cultures as sustainable platforms for high-value lignan production (Liu et al., 2025); (Motolinía-Alcántara et al., 2021); (Verdú-Navarro et al., 2023; Titova et al., 2024)).

#### 7.4.6 Critical comparison between platforms and future perspectives

A comparison of available culture systems demonstrates distinct differences in performance and application. Cell suspension cultures are notable for their high responsiveness to elicitation and their capacity to rapidly alter metabolic pathways, which accounts for the elevated lignan yields frequently observed in these systems. In contrast, hairy root cultures produce some of the highest levels of aryltetralin lignans and are particularly well-suited for metabolic pathways linked to root differentiation. Although callus cultures typically exhibit lower and more variable productivity, they remain important for establishing, selecting, and optimizing producer lines prior to transfer into more productive systems (Berim et al., 2005; Ionkova et al., 2010; Renouard et al., 2018; Morey and Peebles, 2022).

These distinctions underscore a fundamental trade-off between flexibility and stability. Undifferentiated systems, including callus cultures and cell suspensions, are more amenable to manipulation and respond efficiently to culture modifications and elicitation treatments. Conversely, differentiated systems, particularly hairy roots, typically offer greater metabolic stability and sustained lignan production, though they present challenges in establishment and scale-up (Berim et al., 2005; Ionkova et al., 2010; Renouard et al., 2018; Morey and Peebles, 2022).

Despite notable progress, several limitations continue to hinder direct comparisons among studies. Reported yields are frequently expressed in different units, such as dry weight (DW) or fresh weight (FW), and production parameters are not consistently standardized. Additionally, multiple lignans are sometimes aggregated into a single value. Productivity is also strongly affected by species, genotype, and the specific cell line or root clone employed, further complicating cross-study comparisons (Berim et al., 2005; Ionkova et al., 2010; Renouard et al., 2018; Morey and Peebles, 2022).

Future advancements are likely to depend on integrating the strengths of each culture platform. For cell suspensions, further optimization of elicitation strategies using compounds such as chitosan, salicylic acid, methyl jasmonate, and nanoparticles may enhance lignan yields. For hairy roots, efforts should prioritize increasing productivity while improving bioreactor performance and scalability. For callus cultures, the primary objective remains the development of stable and highly productive lines to serve as the foundation for subsequent culture systems (Bazaldúa et al., 2019; Berim et al., 2005; Ionkova et al., 2010; Renouard et al., 2018; Morey and Peebles, 2022).

In summary, no single platform is universally optimal for producing all lignans. Rather, callus cultures, cell suspensions, and hairy roots should be regarded as complementary tools, each offering distinct advantages. Callus cultures facilitate line establishment and selection, cell suspensions enable metabolic optimization and elicitation studies, and hairy roots support stable production of specialized lignans. Integrating these approaches with elicitation strategies, metabolic engineering, and bioprocess optimization is anticipated to further enhance the sustainable production of lignans for pharmaceutical, nutraceutical, and industrial applications (Berim et al., 2005; Ionkova et al., 2010; Renouard et al., 2018; Morey and Peebles, 2022).

## 8 Challenges and perspectives

Lignans represent a structurally diverse group of plant-derived phenolic secondary metabolites with significant physiological roles in plants and notable pharmacological potential. These compounds are synthesized via the phenylpropanoid pathway, a central metabolic route that produces defense, signaling, and structural metabolites. The key step in lignan biosynthesis is the oxidative coupling of monolignols, typically coniferyl alcohol, which is a radical process subject to highly specific stereochemical control, in contrast to lignification. This stereochemical specificity is directed by proteins known as directing proteins (DIRs), which guide radical dimerization toward defined chiral configurations without intrinsic catalytic activity. The establishment of specific chirality is crucial for lignan biological activity and presents a major challenge for total chemical synthesis, as reproducing this stereoselective control artificially is difficult. From a production perspective, isolating lignans from natural plant sources is problematic due to their low concentrations and occurrence in slow-growing or geographically restricted species, such as those in the genera Linum and Podophyllum. This limitation has prompted the development of biotechnological approaches that use *in vitro* plant tissue cultures, thereby separating metabolite production from ecological and seasonal constraints. Commonly employed systems include cell suspensions and, with greater technological robustness, hairy roots or Agrobacterium rhizogenes-transformed roots. These transformed roots offer high genetic stability, autonomous growth, and sustained biosynthetic capacity, making them particularly suitable for the production of structurally complex lignans.

Metabolic elicitation is fundamental for enhancing lignan production in these systems. Controlled application of biotic or abiotic elicitors, including methyl jasmonate, microbial extracts, or physicochemical stress signals, initiates cellular defense responses that activate key enzymes in the phenylpropanoid pathway. This metabolic shift increases carbon flux toward the biosynthesis of specific lignans, such as secoisolariciresinol diglucoside, a precursor for pharmacologically relevant compounds. However, the extent of these responses depends heavily on the culture’s physiological state and the stimulus intensity, leading to yield variability and complicating large-scale standardization.

The physicochemical properties of lignans significantly affect their biological activity and determine appropriate routes of administration. These compounds possess a diphenylpropanoid core, and their functionalization influences polarity, solubility, and stability. In aglycone form, many lignans display moderate lipophilicity, with logP values that facilitate passive membrane permeation. However, this property also results in low aqueous solubility, limiting gastrointestinal dissolution and reducing oral bioavailability. In contrast, glycosylation increases hydrophilicity, enhancing solubility in aqueous environments but decreasing membrane permeability. Consequently, systemic absorption of glycosylated lignans relies on enzymatic hydrolysis by the gut microbiota, leading to substantial interindividual variability and complicating pharmacokinetic management.

The intermediate molecular weight of lignans, combined with their susceptibility to oxidation and first-pass hepatic metabolism, limits the feasibility of alternative routes of administration, such as transdermal or sublingual delivery. These challenges necessitate advanced formulations or structural modifications for clinical use. As a result, semisynthesis has become the predominant pharmaceutical strategy, integrating complex natural precursors with chemical modifications to enhance solubility, stability, and pharmacodynamic properties. Etoposide exemplifies this approach, being suitable for both oral and intravenous administration, although it requires sophisticated formulation technologies.

Despite these advancements, scalability remains a significant challenge. The transition of plant culture systems to industrial bioreactors is impeded by cell sensitivity to hydrodynamic stress, metabolic heterogeneity, and mass-transfer limitations, all of which compromise process reproducibility. Although *in vitro* systems alleviate pressure on wild plant populations, they still involve considerable energy consumption and operational expenses.

In this context, current prospects focus on integrating plant biotechnology with synthetic biology and metabolic engineering to reconstruct lignan biosynthetic pathways in recombinant microorganisms. This strategy offers the potential for more scalable and controllable production processes, though challenges remain regarding enzyme folding, stereochemical control of oxidative coupling, and host metabolic compatibility. Ultimately, sustainable lignan development depends on combining detailed mechanistic understanding, robust production systems, and advanced molecular and pharmaceutical design to realize their biological potential in clinically viable and environmentally responsible applications.
